# Tumor-released autophagosomes induces CD4^+^ T cell-mediated immunosuppression via a TLR2–IL-6 cascade

**DOI:** 10.1186/s40425-019-0646-5

**Published:** 2019-07-12

**Authors:** Yong-Qiang Chen, Peng-Cheng Li, Ning Pan, Rong Gao, Zhi-Fa Wen, Tian-Yu Zhang, Fang Huang, Fang-Yuan Wu, Xi-Long Ou, Jin-Ping Zhang, Xue-Jun Zhu, Hong-Ming Hu, Kang Chen, Yun-Lang Cai, Li-Xin Wang

**Affiliations:** 1 Department of Microbiology and Immunology, Medical School of Southeast University, 87 Dingjiaqiao Road, Nanjing, 210009 China; 2 Department of Obstetrics and Gynecology, Zhongda Hospital, Medical School of Southeast University, 87 Dingjiaqiao Road, Nanjing, 210009 China; 3 Department of Gastroenterology, Zhongda Hospital, Medical School of Southeast University, Nanjing, 210009 China; 4 Robert W. Franz Cancer Research Center, Earle A. Chiles Research Institute, Providence Portland Medical Center, Portland, OR 97213 USA; 5 Institutes of Biology and Medical Sciences, Soochow University, Suzhou, 215123 China; 6 Department of Obstetrics and Gynecology and Barbara Ann Karmanos Cancer Institute, Wayne State University, Mucosal Immunology Studies Team, National Institute of Allergy and Infectious Diseases, National Institutes of Health, Detroit, MI 48201 USA; 7 Jiangsu Province Hospital of Traditional Chinese Medicine, The Affiliated Hospital of Nanjing University of Chinese Medicine, Nanjing, 210029 China

**Keywords:** Extracellular vesicles (EVs), Tumor-released autophagosome (TRAP), CD4^+^ T cell, Regulatory B cell, IL-6, Heat shock protein 90α (HSP90α)

## Abstract

**Background:**

CD4^+^ T cells are critical effectors of anti-tumor immunity, but how tumor cells influence CD4^+^ T cell effector function is not fully understood. Tumor cell-released autophagosomes (TRAPs) are being recognized as critical modulators of host anti-tumor immunity during tumor progression. Here, we explored the mechanistic aspects of TRAPs in the modulation of CD4^+^ T cells in the tumor microenvironment.

**Methods:**

TRAPs isolated from tumor cell lines and pleural effusions or ascites of cancer patients were incubated with CD4^+^ T cells to examine the function and mechanism of TRAPs in CD4^+^ T cell differentiation and function. TRAPs-elicited CD4^+^ T cells were tested for their suppression of effector T cell function, induction of regulatory B cells, and promotion of tumorigenesis and metastasis in a mouse model.

**Results:**

Heat shock protein 90α (HSP90α) on the surface of TRAPs from malignant effusions of cancer patients and tumor cell lines stimulated CD4^+^ T cell production of IL-6 via a TLR2–MyD88–NF-κB signal cascade. TRAPs-induced autocrine IL-6 further promoted CD4^+^ T cells secretion of IL-10 and IL-21 via STAT3. Notably, TRAPs-elicited CD4^+^ T cells inhibited CD4^+^ and CD8^+^ effector T cell function in an IL-6- and IL-10-dependent manner and induced IL-10-producing regulatory B cells (Bregs) via IL-6, IL-10 and IL-21, thereby promoting tumor growth and metastasis. Consistently, inhibition of tumor autophagosome formation or IL-6 secretion by CD4^+^ T cells markedly retarded tumor growth. Furthermore, B cell or CD4^+^ T cell depletion impeded tumor growth by increasing effector T cell function.

**Conclusions:**

HSP90α on the surface of TRAPs programs the immunosuppressive functions of CD4^+^ T cells to promote tumor growth and metastasis. TRAPs or their membrane-bound HSP90α represent important therapeutic targets to reverse cancer-associated immunosuppression and improve immunotherapy.

**Electronic supplementary material:**

The online version of this article (10.1186/s40425-019-0646-5) contains supplementary material, which is available to authorized users.

## Background

CD4^+^ T cells play a critical role in modulating both innate and adaptive anti-tumor immune responses. Research over the past two decades has revealed that CD4^+^ effector T cells, especially IFN-γ-producing T helper 1 (Th1) cells, can exhibit anti-tumor activity [1]. However, other subtypes of tumor-infiltrating CD4^+^ T cells may play a pro-tumorigenic role in the tumor microenvironments via the secretion of inflammatory or regulatory cytokines, such as interleukin (IL)-6, IL-10, IL-17, IL-21, and transforming growth factor (TGF)-β, as the abundance of such CD4^+^ T cells has been associated with a poor clinical outcome of various types of cancer [1–4]. It has also become clear that many tumor-derived molecules or extracellular vesicles likely influence the differentiation of CD4^+^ T cells [5, 6]. However, the precise mechanisms underlying CD4^+^ T cell differentiation and functions in the tumor microenvironment are not completely understood.

Extracellular vesicles (EVs) have emerged as a new mode of intercellular communication by functioning as the carriers of bioactive molecules to influence the extracellular environment and the immune system [6–8]. Recent evidences indicate that secretory autophagy, in contrast to canonical autophagy, is an alternative non-degradative mechanism for cellular trafficking and unconventional secretion of proteins and small molecules [9], such as IL-1β [10], high mobility group box 1 (HMGB1) [11], adenosine triphosphate (ATP) [12], TGF-β [13], and lysozyme [14]. More importantly, secretory autophagosomes carrying cytoplasmic cargoes, including tumor-specific antigens or viruses, fail to fuse with lysosomes and instead are released into the extracellular environment by the cells under stress [15, 16].

We have previously found extracellular secretory autophagosomes from the supernatant of tumor cells or malignant effusions and ascites of cancer patients [17, 18], and have termed such tumor-released autophagosomes TRAPs. We confirmed that TRAPs can be taken up by phagocytes such as neutrophils and macrophages, as well as B cells, and endow them with immunosuppressive activities [18–20]. These observations highlight that TRAPs are part of an elaborate network of tumor-derived vesicles that can reroute the immune response towards a cancer-promoting direction and should be targeted to improve cancer therapy. However, the mechanistic aspects of TRAPs in the modulation of immune cell function, especially the key anti-tumor effector cell, CD4^+^ T cell, in the tumor microenvironment and during tumor progression are unclear.

Here, we demonstrate that TRAPs could educate CD4^+^ T cells to produce IL-6 that functions in an autocrine manner to promote the production of IL-10 and IL-21. TRAPs-elicited CD4^+^ T cells (T_TRAP_) directly inhibit the anti-tumor IFN-γ response of CD4^+^ T and CD8^+^ T cells and also induce IL-10^+^ Bregs, which creates a favorable environment to facilitate tumor growth and metastasis. Mechanistic studies revealed that membrane-bound HSP90α on intact TRAPs is crucial for inducing IL-6 production in CD4^+^ T cells via a TLR2–MyD88–NF-κB signal cascade. Moreover, autocrine IL-6 further stimulates CD4^+^ T cells to produce IL-10 and IL-21 via STAT3. Our study unveils novel cellular and molecular mechanisms of tumor-derived extracellular vesicles in regulating CD4^+^ effector T cell function and pinpoint TRAPs as a therapeutic target for cancer immunotherapy.

## Materials and methods

### Human subjects

Malignant pleural effusions and ascites were collected from cancer patients pathologically diagnosed with multiple cancer types. The clinicopathological characteristics of the enrolled patients are presented in Additional file 1: Table S1. The study was approved by the Ethics Committee for Human Studies of Southeast University (protocol 2016ZDKYSB112).

### Mice

C57BL/6 female mice were purchased from the Comparative Medicine Center of Yangzhou University. *Tlr4*^−/−^, *Tlr2*^−/−^, *Myd88*^−/−^ and OT-I mice were purchased from the Nanjing Biomedical Research Institute of Nanjing University (Nanjing, China). *Il6*^−/−^ mice were gifts from Dr. Jinping Zhang (Institutes of Biology and Medical Sciences, Soochow University, Suzhou, China). Mice were maintained in the barrier facility at Southeast University. All animal experiments were approved by the Institutional Animal Care and Use Committee of Southeast University.

### Cell culture

The murine hepatic carcinoma line Hepa1–6, melanoma line B16F10, Lewis lung carcinoma line LLC, lymphoma line EL4, and the human melanoma line A375, hepatic carcinoma line HepG2 and breast carcinoma line MDA-MB-231 were cultured in complete RPMI-1640 medium with 10% FBS (Gibco), 100 U/ml penicillin and 0.1 mg/ml streptomycin at 37 °C in a 5% CO_2_ incubator. *Becn1* knockdown (*Becn1* KD) and negative control B16F10 cells (*Becn1* NC) were established by using lentivirus expressing *Becn1*-targeting (5′- GCGGGAGUAUA GUGAGUUUTT-3′) and scrambled (5′-TTCTCCGAACGTGTCACGTAA-3′) shRNA (Hanbio Biotechnology, Shanghai, China), respectively.

### Chemicals

The inhibitors PD98059, SP600125, SB203580, LY294002, BAY11–7082, and Stattic were purchased from MCE (Shanghai, China). Recombinant murine IL-2 and IL-12 were purchased from PeproTech (Rocky Hill, USA). CFSE were purchased from Invitrogen/Thermo Fisher Scientific. IL-6, IL-10 and IL-21 neutralizing antibodies were purchased from R&D Systems. Lymphocyte separation media were purchased from MultiSciences (Hangzhou, China). All other reagents were obtained from Sigma-Aldrich (St. Louis, MO).

### TRAPs purification and characterization

Tumor cells were seeded in a T175 flask in complete RPMI-1640 culture medium supplemented with 10% heat-inactivated FBS (Gibco), 100 U/ml penicillin, and 0.1 mg/ml streptomycin and incubated for 3–4 days at 37 °C, 5% CO_2_ until 100% confluency was reached. Tumor cell culture supernatants were collected for TRAPs isolation as described previously [18, 20]. Briefly, supernatants were centrifuged at 2000 rpm for 10 min to remove whole cells and debris. The supernatants were further centrifuged at 12,000 g for 30 min to harvest the TRAPs-containing pellet. The TRAPs-containing pellet was washed three times with PBS and isolated with magnetic beads (Miltenyi Biotec) combined with LC3b antibody (Cell Signaling Technology) for TRAPs. The purity of TRAPs was analyzed by flow cytometry and western blot. The size of TRAPs was determined by dynamic light scattering using a Malvern Instrument.

### Primary cell isolation

Mouse splenic B cells (Invitrogen, 11422D), CD4^+^ T cells (Invitrogen, 11415D), CD8^+^ T cells (Invitrogen, 11417D) and human peripheral blood CD4^+^ T cells (Miltenyi Biotec, 130–045-101) were purified by magnetic-activated cell sorting (MACS) following the manufacturer’s instructions. After the MACS, the purity of T and B cells were > 95% as assessed by flow cytometry.

### Flow cytometry

Purified CD4^+^ T or CD8^+^ T cells were cultured in a 24-well plate pre-coated with 2 μg/ml anti-CD3 (BD Biosciences, 550,275) and 2 μg/ml anti-CD28 mAb (BD Biosciences, 553,294) in the presence of 50 U/ml IL-2 (PeproTech), purified TRAPs and 30% culture supernatants from CD4^+^ T cells or B cells. In some cases, culture supernatants from CD4^+^ T cells or B cells were pretreated with neutralizing mAbs against IL-6, IL-10, or IL-21 for 1 h at 4 °C and subsequently exposed to T cells or B cells. Three days later, IFN-γ^+^ CD4^+^ T, IFN-γ^+^ CD8^+^ T or IL-10^+^ B cells were evaluated by flow cytometry. For intracellular staining, the cells were stimulated with the ovalbumin (OVA) protein or anti-CD3 and anti-CD28 mAbs at 37 °C for 24 or 72 h. Leukocyte activation cocktail and GolgiPlug (BD Biosciences) were added to the culture 5 h prior to flow cytometric analysis. Subsequently, the cells were stained with antibodies specific to the various surface molecules, fixed and permeabilized with a Fixation/Permeabilization Kit (BD Biosciences), and finally stained with antibodies against the various intracellular molecules. To detect Bcl-6 and Foxp3, the cells were fixed and permeabilized using a Transcription Factor Buffer Set (BD Biosciences). Data were acquired using a FACS Calibur analyzer (BD Biosciences) and analyzed by FlowJo. The gates were set according to the staining by isotype-matched control antibodies of the respective cells. The fluorochrome-conjugated Abs used are listed in Additional file 1: Table S2.

### Quantitative real-time PCR

Total RNA from CD4^+^ T cells was isolated with TRIzol reagent (Invitrogen) and reverse-transcribed using 5 × PrimeScriptRT Master Mix (Takara), following the manufacturer’s instructions. The specific primers used to amplify the genes are listed in Additional file 1: Table S3. The PCR was performed in triplicate using Fast Start Universal SYBR Green Master (ROX) (Roche Life Science) in a StepOne Real-Time PCR System (Thermo Fisher Scientific). GAPDH was used as an internal standard.

### Elisa

Cytokines in the sera or cell culture supernatants were quantified using ELISA kits according to the manufacturer’s protocol. ELISA sets were purchased from eBioscience (IL-6 and IL-10) and R&D Systems (IL-21).

### Western blot

The proteins samples were extracted from CD4^+^ T cells with RIPA lysis buffer. They were separated and transferred as previously described [21]. The membranes were blocked with 5% BSA in TBST for 1 h and separately incubated with the primary antibodies overnight at 4 °C. After washing with TBST buffer, the membranes were incubated with horseradish peroxidase-conjugated secondary antibodies for 1 h. The results were visualized by enhanced chemiluminescence according to the manufacturer’s protocol. The primary antibodies used are listed in Additional file 1: Table S4.

### Animal models

Wild type C57BL/6 mice or *Il6*^−/−^ C57BL/6 mice were subcutaneously inoculated with B16F10, B16F10 *Becn1* NC or B16F10 *Becn1* KD cells (2 × 10^5^ cells/mouse). Tumor growth was measured using a caliper. On day 21, draining lymph nodes (dLN), spleens or tumor tissues were harvested from tumor-free or tumor-bearing mice. The frequencies of IL-10^+^ CD4^+^ T cells, IL-21^+^ CD4^+^ T cells, or IL-10^+^ B cells were evaluated by flow cytometry after ex vivo stimulation with the leukocyte activation cocktail and GolgiPlug (BD Biosciences) for 5 h. In the subcutaneous tumor model, B16F10 tumor cells (2 × 10^5^ cells/mouse) and CD4^+^ T cells treated with TRAPs, or B cells treated with the indicated culture conditions (2 × 10^6^ cells/mouse) were subcutaneously injected into the right flank of C57BL/6 mice. Subcutaneous tumor growth was monitored and measured using vernier calipers. In the tumor metastasis model, B16F10 tumor cells (5 × 10^5^ cells/mouse) were intravenously injected into C57BL/6 mice and TRAPs-treated or untreated CD4^+^ T cells or B cells (5 × 10^6^ cells/mouse) treated with the indicated culture conditions were injected every other day for 3 times. Three weeks later, mice were sacrificed, and the tumor nodules in the lungs were examined. To evaluate the role of CD4^+^ T cells and B cells treated with the indicated culture conditions in OVA-loaded DC_−_mediated specific immune response, C57BL/6 mice were adoptively transferred with OT-I splenocytes (1 × 10^7^ cells/mouse) on day 0 and vaccinated with OVA-loaded DCs (1 × 10^6^ cells/mouse) on days 1, 4, and 7. After intravenous administration of CD4^+^ T cells and B cells on days 2, 5, and 8, mice from each group were sacrificed on day 14 and the frequency and number of CD8^+^Vβ5.1^+^ T cells were evaluated by flow cytometry. The frequency of IFN-γ^+^ CD4^+^ and CD8^+^ T cells in the spleens was determined by intracellular cytokine staining after ex vivo stimulation with the OVA protein for 24 h.

### T and B cell depletion

C57Bl/6 mice (*n* = 5/group) were inoculated subcutaneously in the flank with 1 × 10^6^ *Becn1*-NC or *Becn1-*KD B16F10 cells. On day 9, the tumor-bearing mice were subsequently depleted of either CD4^+^ T cells, CD8^+^ T cells or CD20^+^ B cells by intravenous administration of 250 μg/mouse of anti-mouse CD4 (clone GK1.5, BioXCell) or anti-mouse CD8 (clone 2.43, BioXCell) twice weekly throughout the course of tumor growth, or 250 μg of anti-mouse CD20 (clone SA271G2, BioLegend), respectively. Control mice were treated similarly but with isotype-matched control antibodies. Depletion was confirmed by staining of peripheral blood cells with anti-mouse CD4 (RM4–5, BD Pharmingen), anti-mouse CD8 (clone 53–6.7, BioLegend), or anti-mouse CD19 (clone 6D5, BioLegend).

### Statistical analysis

Data were derived from at least 3 independent experiments and analyzed using GraphPad Prism 5.0 software. Multiple group comparisons were performed by one-way ANOVA and the Tukey-Kramer multiple test. Comparisons between 2 groups were performed using unpaired Student’s t-test or Mann-Whitney U test. *P* < 0.05 was considered significant.

## Results

### TRAPs induce CD4^+^ T cells to produce IL-6, IL-10, and IL-21

**Fig. 1:**
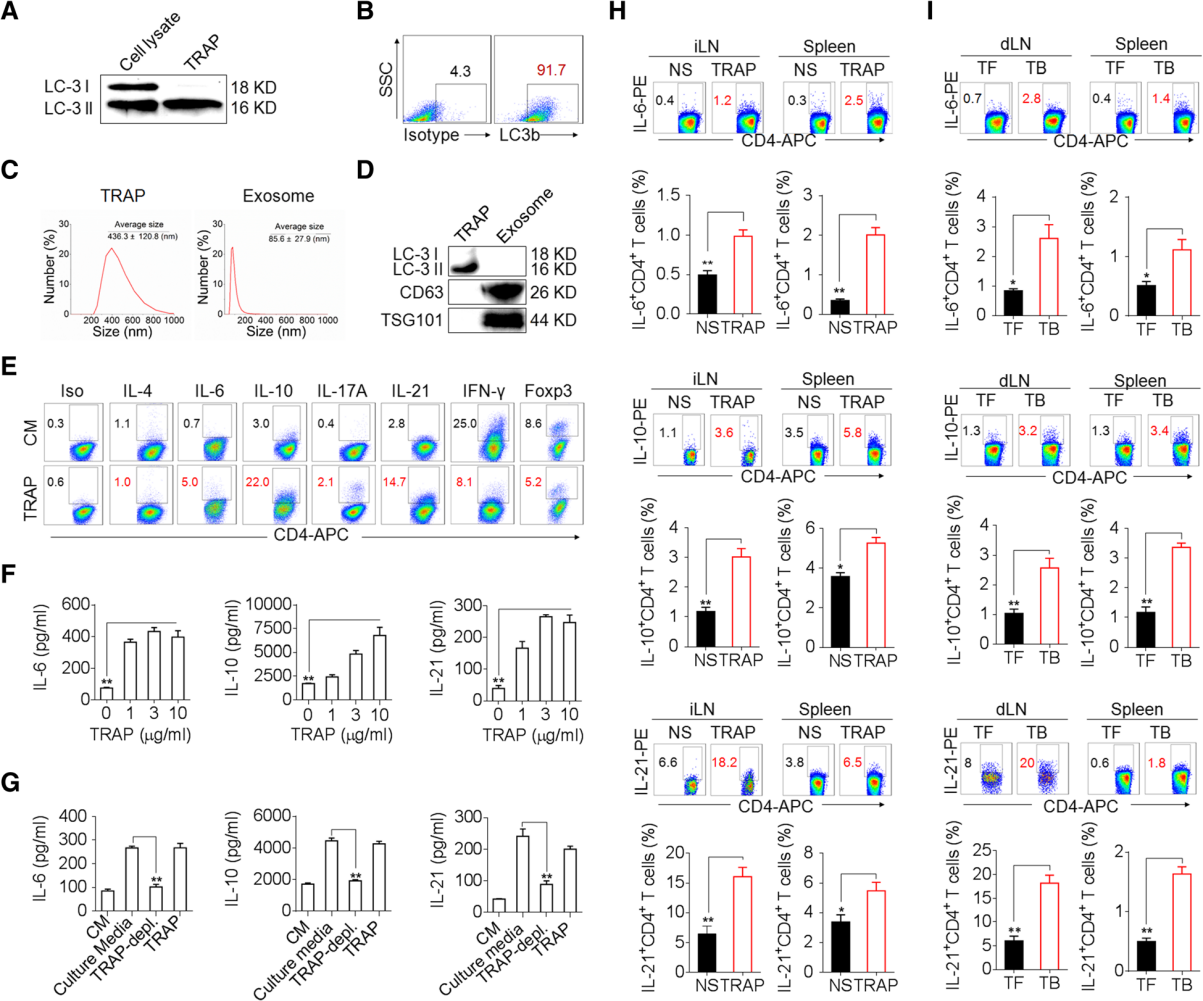
TRAPs induce IL-6, IL-10, and IL-21 expression by CD4^+^ T cells. **a–d** Characterization of TRAPs from B16F10 cells, including Western blot (**a**, **d**), flow cytometric (**b**), and dynamic light scattering (**c**) analyses of the expression of the autophagosome-specific marker LC-3II, the percentage, and the size distribution of the isolated TRAPs. **e** Flow cytometric determination of the percentage of IL-6-, IL-21-, IL-10-, IL-4-, IL-17-, IFN-γ-, and Foxp3-expressing CD4^+^ T cells treated with control media (CM) or 3 μg/ml TRAPs in the presence of anti-CD3 and anti-CD28 for 72 h. **f** ELISA of IL-6, IL-10, and IL-21 secretion by CD4^+^ T cells treated with CM or 1, 3, or 10 μg/ml TRAPs in the presence of anti-CD3 and anti-CD28 for 72 h. **g** ELISA of IL-6, IL-10 and IL-21 secretion by CD4^+^ T cells cultured in B16F10 tumor cell-conditioned culture media, TRAP-depleted tumor cell culture media or TRAPs purified from the equal cell culture media in the presence of anti-CD3 and anti-CD28 for 72 h. **h** Flow cytometric and statistical analyses of the percentage of IL-6^+^, IL-10^+^ or IL-21^+^ CD4^+^ T cells in the inguinal lymph nodes (iLN) and spleens of C57BL/6 mice (*n* = 6) 7 days after i.v. administration of normal saline (NS) or TRAPs (30 μg/mouse) every other day for 3 times. **i** Flow cytometric and statistical analyses of the percentage of IL-6^+^, IL-10^+^ or IL-21^+^ CD4^+^ T cells in the draining lymph nodes (dLN) and spleens of C57BL/6 tumor-bearing (TB) mice (*n* = 6) 21 days after s.c. inoculation of B16F10 cells, in comparison to the tumor-free (TF) mice. Data (mean ± SEM) represent 3 independent experiments. *, *P* < 0.05; **, *P* < 0.01; ***, *P* < 0.001; ns, not significant, by one-way ANOVA with the Tukey-Kramer multiple test, 2-tailed unpaired t-test or Mann-Whitney U test

To determine whether TRAPs impact CD4^+^ T cell function, we first isolated TRAPs from the culture supernatants of mouse B16F10 melanoma cells [18, 19]. The TRAPs preparation specifically contained the mature autophagosome marker LC-3II (Fig. 1a, b) and exhibited an average size of 436.3 nm, which was distinct from isolated exosomes that had an average size of 85.6 nm (Fig. 1c) and expressed the exosome markers CD63 and TSG101 (Fig. 1d). Treatment of mouse splenic CD4^+^ T cells with TRAPs during activation by anti-CD3 and anti-CD28 resulted in the induction of the transcripts encoding *Il6*, *Il21*, *Il10*, and *Il17*, but not *Il1b*, *Il2*, *Il4*, *Il9*, *Tnf*, *Ifng*, *Foxp3* or *Tgfb1* (Additional file 2: Figure S1a). Consistently, the frequency of IL-6^+^, IL-10^+^ or IL-21^+^ CD4^+^ T cells and the secretion of IL-6, IL-10 or IL-21 by CD4^+^ T cells were increased by TRAPs treatment (Fig. 1e, f). TRAPs-induced IL-21^+^ CD4^+^ T cells expressed elevated levels of the follicular helper T cell (Tfh)-associated molecules CXCR5 and Bcl-6 (Additional file 2: Figure S1b, c). In contrast, TRAPs reduced the frequency of IFN-γ^+^ CD4^+^ T cells (Fig. 1e) and suppressed IL-12-mediated induction of IFN-γ^+^ Th1 cells (Additional file 2: Figure S1d). Depletion of TRAPs from the culture media via ultracentrifugation (Additional file 2: Figure S1e) resulted in a significant reduction of IL-6, IL-10 and IL-21 production by CD4^+^ T cells (Fig. 1g). Intriguingly, we also found that LC3B^+^ EVs (TRAPs) purified from B16F10 culture supernatant were more potent than LC3B^−^ EVs and exosomes in upregulating IL-6 expression, suggesting that LC3B^+^ EVs (TRAPs) are the dominant large EVs that instruct CD4^+^ T cells (Additional file 2: Figure S2a-d). In order to ascertain the role of TRAPs in inducing IL-6, IL-10 and IL-21 production by CD4^+^ T cells in vivo, normal saline (NS) or TRAPs were administered intravenously (i.v.) into C57BL/6 mice every other day for 3 times. The frequencies of IL-6^+^, IL-10^+^ and IL-21^+^ CD4^+^ T cells in the inguinal lymph node and spleen increased markedly after TRAPs administration (Fig. 1h). Consistently, in B16F10 tumor-bearing mice, the frequency of IL-6^+^, IL-21^+^ and IL-10^+^ CD4^+^ T cells in the draining lymph node and spleen were also increased (Fig. 1i). Taken together, these results show that TRAPs could modulate CD4^+^ T cell differentiation by inducing IL-6, IL-10, and IL-21 expression and suppressing their IFN-γ production.

### TRAPs-induced IL-6, IL-10, and IL-21 production requires TLR2–MyD88 signaling

**Fig. 2:**
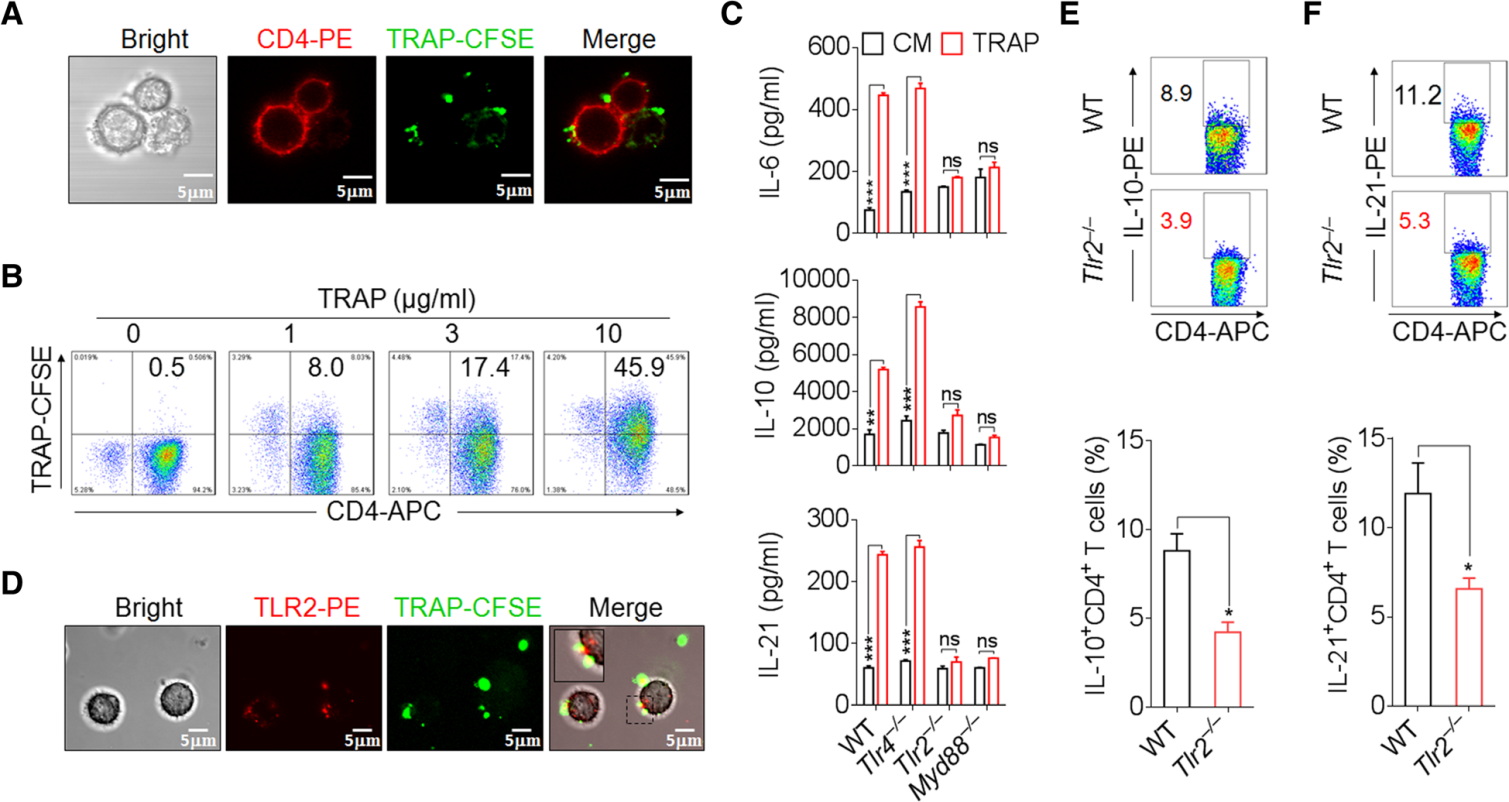
TRAPs induce IL-6/IL-10/IL-21 production of CD4^+^ T cells via TLR2-MyD88 pathway. **a** Confocal microscopy analysis of CFSE-labeled TRAPs (3 μg/ml) and mouse splenic CD4^+^ T cells (stained with anti-CD4-PE) after 24 h of co-culture. Scale bar: 5 μm. **b** Flow cytometric determination of the proportion of CFSE^+^ CD4^+^ T cells after incubated with CFSE-labeled TRAPs (0, 1, 3, or 10 μg/ml) in the presence of anti-CD3 and anti-CD28 for 24 h. **c** ELISA of IL-6, IL-10, and IL-21 secretion by WT, *Tlr2*^−/−^, *Tlr4*^−/−^ or *Myd88*^−/−^ CD4^+^ T cells treated with TRAPs (3 μg/ml) or control media (CM) in the presence of anti-CD3 and anti-CD28 for 72 h. **d** Purified CD4^+^ T cells were co-cultured with CFSE-labeled TRAPs (3 μg/ml) for 24 h, and then stained for TLR2 and analyzed by confocal microscopy. **e, f** Flow cytometric and statistical analyses of the percentage of IL-10^+^ CD4^+^ T cells (e) or IL-21^+^ CD4^+^ T cells (f) in the tumor tissues of WT or *Tlr2*^−/−^ C57BL/6 mice (*n* = 6) 21 days after s.c. inoculation of B16F10 cells. Data (mean ± SEM) represent 3 independent experiments. *, *P* < 0.05; **, *P* < 0.01; ***, *P* < 0.001; ns, not significant, by 2-tailed unpaired t-test or Mann-Whitney U test

We then investigated the mechanism by which TRAPs induce IL-6, IL-10, and IL-21 in CD4^+^ T cells. Within the time frame of the induction of these cytokines, TRAPs adhered to the surface of CD4^+^ T cells in a dose-dependent manner without being internalized (Fig. 2a, b), suggesting the involvement of surface molecules on TRAPs that interact with receptors on CD4^+^ T cells. TRAPs are enriched with various danger-associated molecular patterns (DAMPs) capable of stimulating pattern recognition receptors (PRRs) [17, 18]. CD4^+^ T cells expressed appreciable levels of TLR2 and TLR4 (Additional file 2: Figure S3a). We therefore examined whether TLR2 or TLR4 was involved in sensing TRAPs by CD4^+^ T cells. While TRAPs-induced IL-6, IL-10 and IL-21 secretion by CD4^+^ T cells was independent of TLR4, *Tlr2*^−/−^ and *Myd88*^−/−^ CD4^+^ T cells were completely defective in producing these cytokines in response to TRAPs (Fig. 2c). Consistently, TLR2 on the surface of CD4^+^ T cells was in direct contact with TRAPs (Fig. 2d). In agreement with the above finding, *Tlr2*^−/−^ mice bearing B16F10 tumors had a significant reduction of IL-21^+^ and IL-10^+^ CD4^+^ T cells in the tumor tissue compared to WT tumor-bearing mice (Fig. 2e, f). Collectively, these results show that TRAPs induce CD4^+^ T cells to produce IL-6, IL-10, and IL-21 in a TLR2- and MyD88-dependent manner.

### TRAPs-elicited IL-6 production by CD4^+^ T cells depends on NF-κB/p38/Akt signaling

**Fig. 3:**
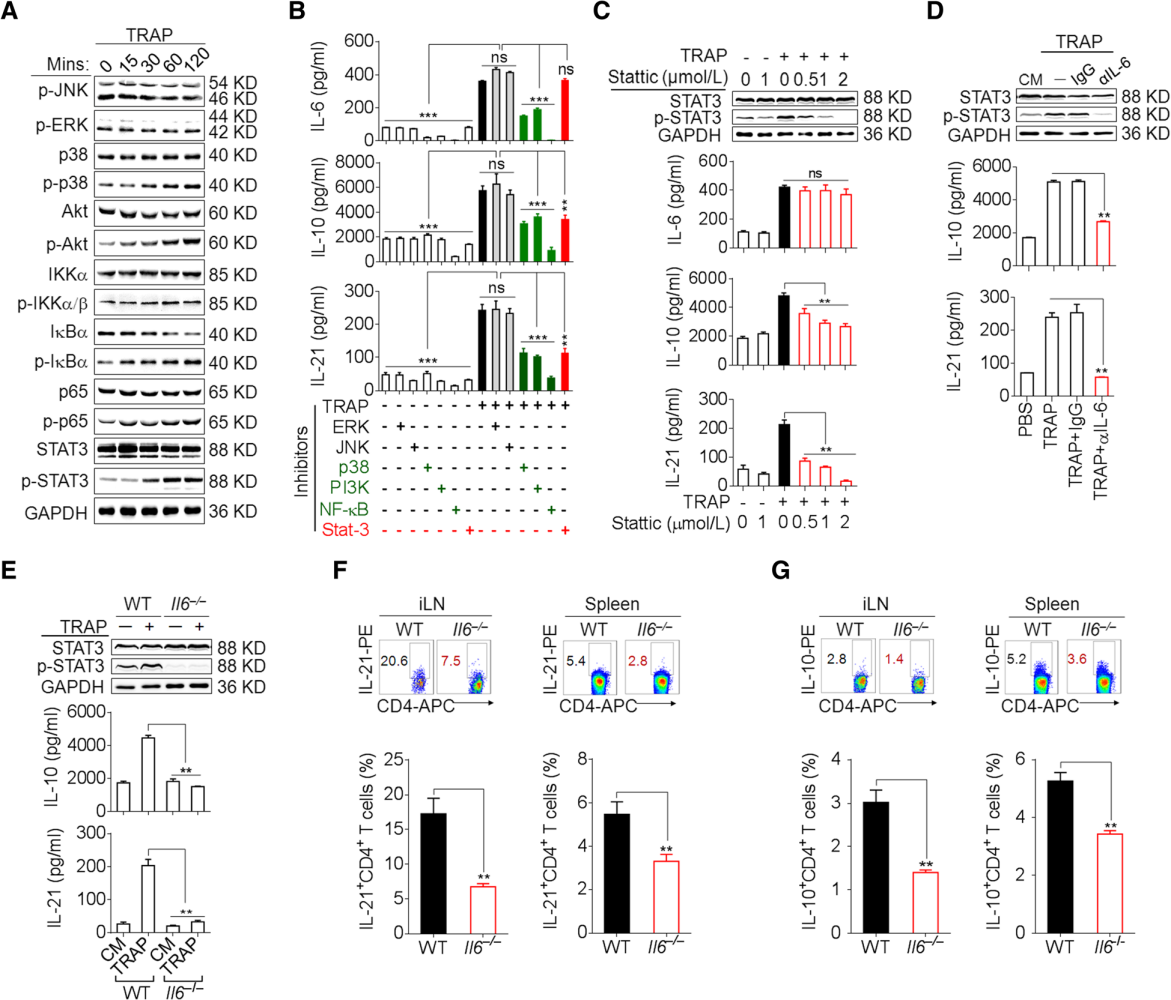
TRAPs promote CD4^+^ T cell expression of IL-6 in an NF-κB/p38/Akt-dependent manner and induce IL-10 and IL-21 via IL-6–STAT3 signaling. **a** Western blot analyses of the phosphorylation of JNK, ERK, p38, Akt, IKKα/β, IκBα, p65 and STAT3 in CD4^+^ T cells treated with TRAPs (3 μg/ml) for the indicated time. **b** CD4^+^ T cells pre-treated with the indicated inhibitors for 1 h and then co-cultured with TRAPs (3 μg/ml) for 72 H*. IL*-6, IL-10 and IL-21 levels in the supernatants were determined by ELISA. **c** Western blot analyses of the phosphorylation of STAT3 in CD4^+^ T cells pre-treated with the STAT3 inhibitor Stattic at the indicated concentrations (0.5, 1 or 2 μM) for 1 h, and then co-cultured with TRAPs (3 μg/ml) for 2 h. ELISA of IL-10 and IL-21 secretion by CD4^+^ T cells treated as above for 72 h. **d** Western blot analyses of the phosphorylation of STAT3 in CD4^+^ T cells treated with anti-IL-6 neutralizing antibody (1 μg/ml) and TRAPs (3 μg/ml) for 2 h. ELISA of IL-10 and IL-21 secretion by CD4^+^ T cells treated as above for 72 h. **e** Western blot analyses of the phosphorylation of STAT3 in WT or *Il6*^−/−^ CD4^+^ T cells treated with TRAPs (3 μg/ml) for 2 h and ELISA of IL-10 and IL-21 secretion by WT or *Il6*^−/−^ CD4^+^ T cells for 72 h. **f, g** Flow cytometric and statistical analyses of the percentage of IL-21^+^ CD4^+^ T cells (f) or IL-10^+^ CD4^+^ T cells (g) in the iLN and spleens of WT or *Il6*^−/−^ C57BL/6 mice (*n* = 6) 7 days after i.v. administration of normal saline (NS) or TRAPs (30 μg/mouse) every other day for 3 times. Data (mean ± SEM) represent 3 independent experiments. *, *P* < 0.05; **, *P* < 0.01; ***, *P* < 0.001; ns, not significant by one-way ANOVA with the Tukey-Kramer multiple test, 2-tailed unpaired t-test or Mann-Whitney U test

We further sought to determine the signals downstream of TLR2 in the induction of IL-6, IL-10, and IL-21 by TRAPs. TRAPs treatment of WT CD4^+^ T cells resulted in the phosphorylation of NF-κB, Akt, p38 and STAT3, but not ERK1/2 or JNK1/2 (Fig. 3a), whereas TRAPs failed to induce NF-κB, Akt, p38 and STAT3 phosphorylation in *Tlr2*^−/−^ or *Myd88*^−/−^ CD4^+^ T cells when compared to CD4^+^ T cells from WT or *Tlr4*^−/−^ mice (Additional file 2: Figure S3b). Pretreatment of CD4^+^ T cells with an inhibitor of NF-κB, Akt or p38 attenuated TRAPs-induced secretion of IL-6, IL-10 and IL-21, whereas the inhibition of JNK1/2 or ERK1/2 had no effect (Fig. 3b). Of note, pretreatment of CD4^+^ T cells with a STAT3 inhibitor diminished the production of IL-10 and IL-21, but not IL-6, in a dose-dependent manner (Fig. 3b, c), indicating that NF-κB, Akt, and p38 activation was needed for TRAPs-induced IL-6, IL-10 and IL-21 production but STAT3 activation was only required for IL-10 and IL-21 production.

### The induction of IL-10 and IL-21 depends on autocrine IL-6 signaling

The IL-6–STAT3 pathway plays a crucial role in Th cell differentiation [22]. Upon IL-6 neutralization with a blocking antibody, the induction of IL-21 and IL-10 mRNA and proteins by TRAPs was completely abolished, with a concomitant decline of STAT3 phosphorylation (Fig. 3d, Additional file 2: Figure S4a). Consistently, TRAPs failed to induce IL-10 and IL-21 expression or STAT3 phosphorylation in *Il6*^−/−^ CD4^+^ T cells (Fig. 3e, Additional file 2: Figure S4b). Moreover, following i.v. administration of TRAPs, the frequencies of IL-10^+^ and IL-21^+^ CD4^+^ T cells in the inguinal lymph node and spleen were much lower in *Il6*^−/−^ mice than in WT mice (Fig. 3f, g). Collectively, these results support a TRAPs-initiated regulatory cascade of CD4^+^ T cell differentiation involving TLR2–NF-κB/p38/Akt-dependent induction of autocrine IL-6 which then promotes IL-10 and IL-21 expression via STAT3.

### Hsp90α is a TRAPs surface ligand that induces IL-6 in CD4^+^ T cells

**Fig. 4:**
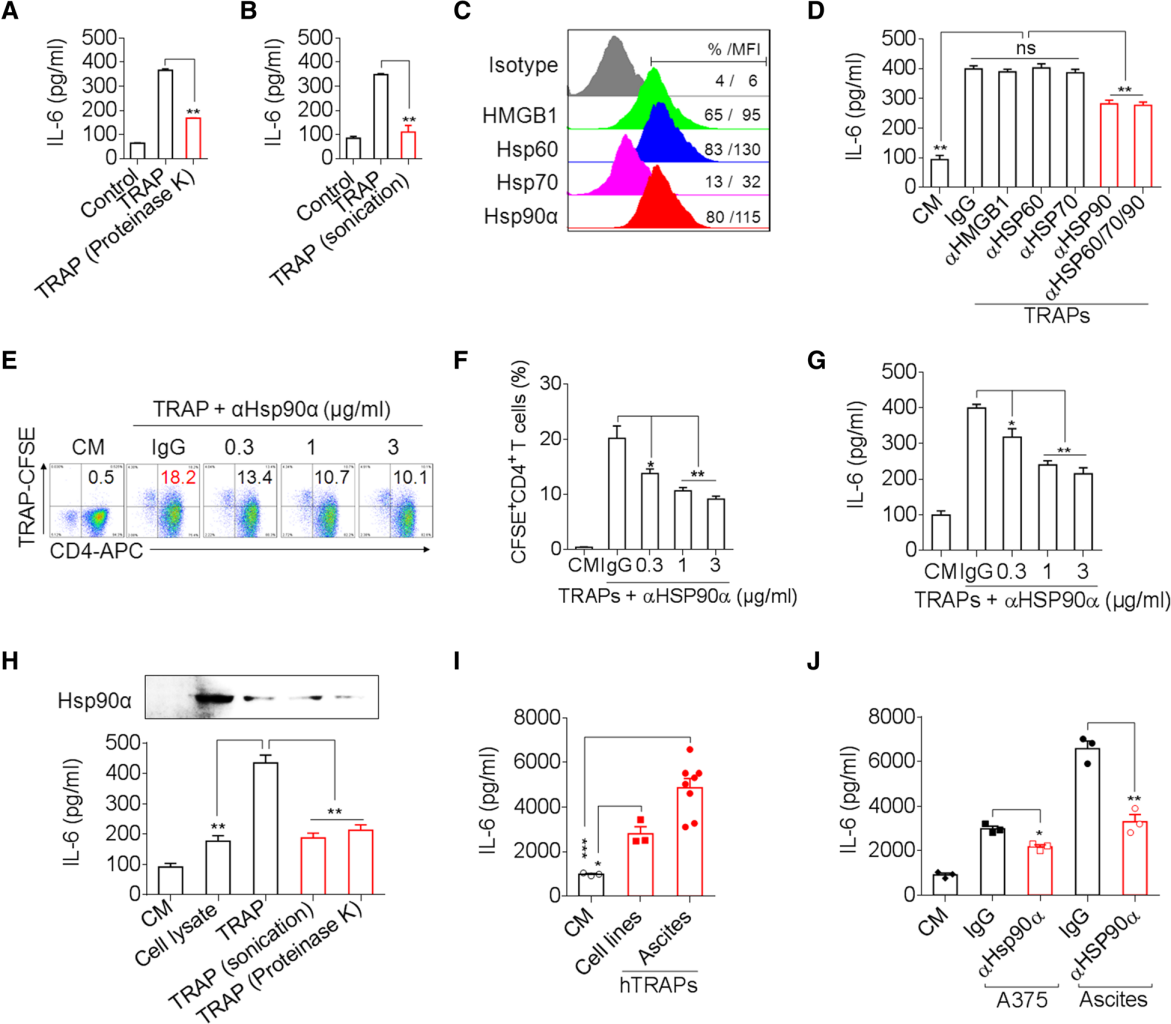
Hsp90α on intact TRAPs is essential for IL-6 induction in CD4^+^ T cells. **a, b** ELISA of IL-6 secretion by CD4^+^ T cells treated with TRAPs (3 μg/ml), proteinase K-digested TRAPs (**a**) or sonicated TRAPs (**b**) for 72 h in the presence of anti-CD3/CD28. **c** Flow cytometric determination of the Hsp60, Hsp70, Hsp90α, or HMGB1 expression levels on the surface of TRAPs from B16F10 tumor cells. **d** ELISA of IL-6 secretion by CD4^+^ T cells treated with TRAPs (3 μg/ml) or blocking antibody-pretreated TRAPs (anti-HMGB1, anti-Hsp60, anti-Hsp70, anti-Hsp90α antibodies) for 72 h in the presence of anti-CD3/CD28. **e-g** CFSE-labeled TRAPs were pretreated with the indicated dose of functional an anti-Hsp90α antibody or an isotype-matched control antibody overnight at 4 °C and then co-cultured with purified CD4^+^ T cells in the presence of anti-CD3/CD28. Twenty-four hour later, the percentage of CFSE^+^ CD4^+^ T cells was assessed by flow cytometry (**e**, **f**). Seventy-two hour later, IL-6 levels in supernatants were determined by ELISA (**g**). **h** The Hsp90α level in tumor cell lysates, an equal amount of TRAPs, sonicated TRAPs, and proteinase K-pretreated TRAPs was determined by western blot. Purified CD4^+^ T cells were co-cultured with the above stimulators for 72 H*. IL*-6 levels in the supernatants were determined by ELISA. **i** ELISA of IL-6 secretion by human CD4^+^ T cells treated with 3 μg/ml human TRAPs (hTRAPs) from 3 human tumor cell lines (A375, MDA-MB-231 and HepG2 cells) or 8 tumor patient effusions and ascites, respectively for 72 h in the presence of anti-CD3/CD28. **j** ELISA of IL-6 secretion by human CD4^+^ T cells treated with hTRAPs (3 μg/ml) or anti-Hsp90α-pretreated hTRAPs for 72 h in the presence of anti-CD3/CD28. Data (mean ± SEM) represent 3 independent experiments. *, *P* < 0.05; **, *P* < 0.01; ns, not significant by one-way ANOVA with the Tukey-Kramer multiple test

To identify the molecular components in TRAPs that are responsible for stimulating CD4^+^ T cell production of IL-6, we first subjected TRAPs to proteinase K digestion or sonication. These treatments impaired the ability of TRAPs to induce IL-6 from CD4^+^ T cells (Fig. 4a, b), indicating that proteins on the surface, but not the soluble contents, of TRAPs are largely responsible for IL-6 induction in CD4^+^ T cells. In addition, TRAPs from the hepatic carcinoma Hepa1–6, lung cancer LLC or lymphoma EL4 cells also potently enhanced IL-6 secretion in CD4^+^ T cells (Additional file 2: Figure S5a). Several ligands of TLR2, including HMGB1, Hsp60, Hsp70, and Hsp90α [18, 23], were enriched in and present on the surface of TRAPs (Fig. 4c, Additional file 2: Figure S5b). Blocking of Hsp90α, but not HMGB1, Hsp60 or Hsp70, on the surface of TRAPs partially diminished TRAPs-induced IL-6 secretion by CD4^+^ T cells, indicating that other molecules on TRAPs may also play a role (Fig. 4d). Accordingly, an anti-Hsp90α antibody dose-dependently inhibited TRAPs binding to CD4^+^ T cells (Fig. 4e, f), reduced TRAPs-induced IL-6 secretion (Fig. 4g), and suppressed the activation of NF-κB, Akt and p38 (Additional file 2: Figure S5c). Remarkably, compared to intact TRAPs, tumor cell lysates containing an equal amount of total protein but much more Hsp90α, or sonicated TRAPs containing an equal amount of Hsp90α, or proteinase K-treated TRAPs were much less effective in inducing IL-6 secretion from CD4^+^ T cells (Fig. 4h). Taken together, these results show that membrane-bound Hsp90α on intact TRAPs effectively induces IL-6 expression from CD4^+^ T cells.

To further determine whether human TRAPs (hTRAPs) could induce human CD4^+^ T cells to produce IL-6, we collected hTRAPs from the culture media of 3 human tumor cell lines, A375, MDA-MB-231 and HepG2, and from the malignant effusions or ascites of 8 cancer patients (Additional file 1: Table S1). Western blotting analysis revealed that LC3-II was expressed at high levels in the collected hTRAPs and Hsp90α was detected in most of hTRAPs (Additional file 2: Figure S5d). RT-PCR analysis and ELISA showed that hTRAPs from cancer patients and tumor cell lines efficiently induced human peripheral blood CD4^+^ T cells to express *IL6* transcript and secrete IL-6 (Fig. 4i, Additional file 2: Figure S5e). Similar to mouse TRAPs, hTRAPs-induced IL-6 transcription and secretion by human CD4^+^ T were almost completely abolished by pretreatment of hTRAPs with an anti-hsp90α blocking antibody (Fig. 4j, Additional file 2: Figure S5f**)**. Altogether, these results indicate that induction of CD4^+^ T cells IL-6 expression by HSP90α on the surface of TRAPs is a common characteristic in humans and mice.

### TRAPs-elicited CD4^+^ T cells (T_TRAP_) suppress effector T cells and promote tumorigenesis

**Fig. 5:**
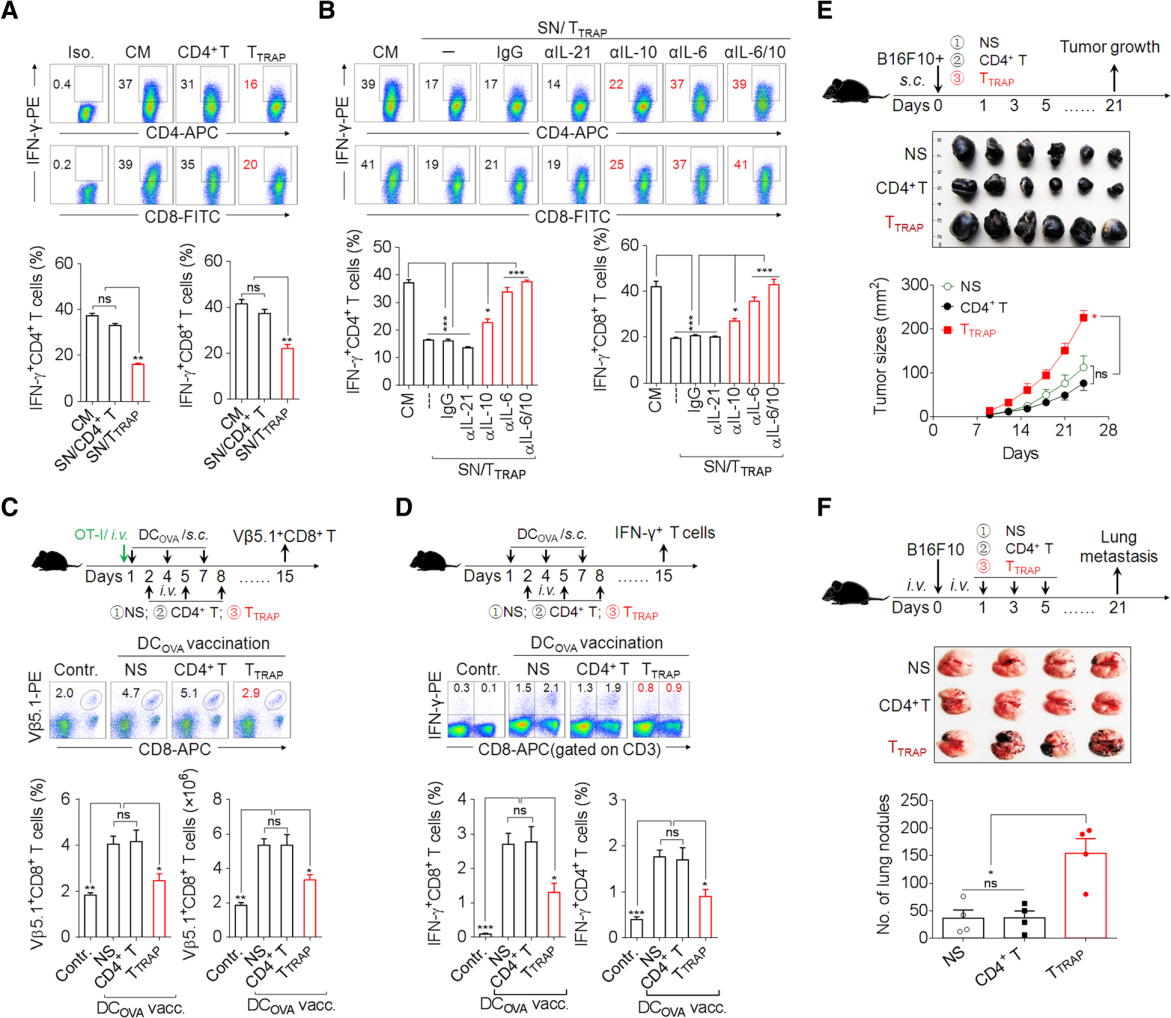
IL-6/IL-10 from T_TRAP_ is responsible for tumor growth and metastasis. **a** Flow cytometric and statistical analyses of the percentage of IFN-γ^+^ CD4^+^ and CD8^+^ T cells treated with the supernatants from T_TRAP_ (SN/T_TRAP_) or control CD4^+^ T cells for 3 d in the presence of anti-CD3/CD28. **b** SN/T_TRAP_ was pretreated with anti-IL-6, IL-10, IL-21 neutralizing antibodies respectively, and then co-cultured with the purified CD4^+^ T cells and CD8^+^ T cells in the presence of anti-CD3/CD28 for 3 d. The percentages of IFN-γ^+^ CD4^+^ T cells and CD8^+^ T cells were evaluated by flow cytometry. **c** C57BL/6 mice were adoptively transferred i.v. with OT-I spleen cells and then vaccinated s.c. with OVA-loaded DC on day 1, 4 and 7, following injection i.v. with T_TRAP_ or control CD4^+^ T cells on day 2, 5 and 8. On the 15th day, the frequencies and the number of Vβ5.1^+^CD8^+^ T cells in spleen were analyzed by flow cytometry. **d** C57BL/6 mice were vaccinated with OVA-loaded DC and following adoptively transferred with T_TRAP_ or CD4^+^ T cells. On the 15th day, the splenocytes were re-stimulated with OVA-protein for 24 h, and the frequencies of IFN-γ^+^ T cells were determined by flow cytometry. **e** B16F10 tumor cells were mixed with T_TRAP_ or control CD4^+^ T cells and injected s.c. into C57BL/6 mice (*n* = 6 per group). The growth of the tumor was monitored. **f** B16F10 tumor cells were intravenously injected into C57BL/6 mice (*n* = 4 to 6 per group) to establish a lung metastasis model. Subsequently, T_TRAP_ or control CD4^+^ T cells were adoptively transferred i.v. 3 times with 1 d of interval. Three weeks later, the tumor nodules in the lungs were examined. Data (mean ± SEM) represent 3 independent experiments. *, *P* < 0.05; **, *P* < 0.01; ***, *P* < 0.001; ns, not significant, by 1-way ANOVA with the Tukey-Kramer multiple test, 2-tailed unpaired t-test or Mann-Whitney U test

To characterize the function of TRAPs-elicited CD4^+^ T cells (T_TRAP_), we activated CD4^+^ and CD8^+^ T cells with anti-CD3 and anti-CD28 in culture supernatants harvested from T_TRAP_ or control CD4^+^ T cells. T_TRAP_ supernatants (SN/T_TRAP_) strongly suppressed the secretion of IFN-γ by activated CD4^+^ and CD8^+^ T cells (Fig. 5a). Pretreatment of SN/T_TRAP_ with a neutralizing antibody against IL-6 or IL-10, but not IL-21, abolished its suppressive effect on IFN-γ production by activated CD4^+^ and CD8^+^ T cells (Fig. 5b). We then transferred control CD4^+^ T cells or T_TRAP_ into C57BL/6 mice that had received OVA-specific Vβ5.1^+^CD8^+^ OT-I T cells and vaccinated with OVA-loaded dendritic cells (DC_OVA_). DC_OVA_ vaccination induced the expansion of Vβ5.1^+^CD8^+^ OT-I T cells in the host, which was suppressed by the adoptive transfer of T_TRAP_ but not control CD4^+^ T cells (Fig. 5c). Moreover, the transfer of T_TRAP_ but not control CD4^+^ T cells led to a decrease of IFN-γ^+^ CD8^+^ and CD4^+^ T cells induced by DC_OVA_ vaccination (Fig. 5d). Therefore, T_TRAP_ could suppress T cell IFN-γ response in vivo.

To see whether T_TRAP_ have a tumor-promoting effect in vivo, we subcutaneously (s.c.) inoculated B16F10 melanoma cells into C57BL/6 mice with or without co-administration of control CD4^+^ T cells or T_TRAP_. Co-administration of B16F10 cells with T_TRAP_ enhanced tumor growth as compared to inoculation of B16F10 cells alone or co-administration with control CD4^+^ T cells (Fig. 5e). When B16F10 melanoma cells were inoculated i.v. together with T_TRAP_, T_TRAP_ promoted tumor metastasis to the lung (Fig. 5f). Collectively, these results show that T_TRAP_ could promote tumor growth and metastasis in vivo.

### T_TRAP_ enhance regulatory B cell function via IL-6, IL-10, and IL-21

**Fig. 6:**
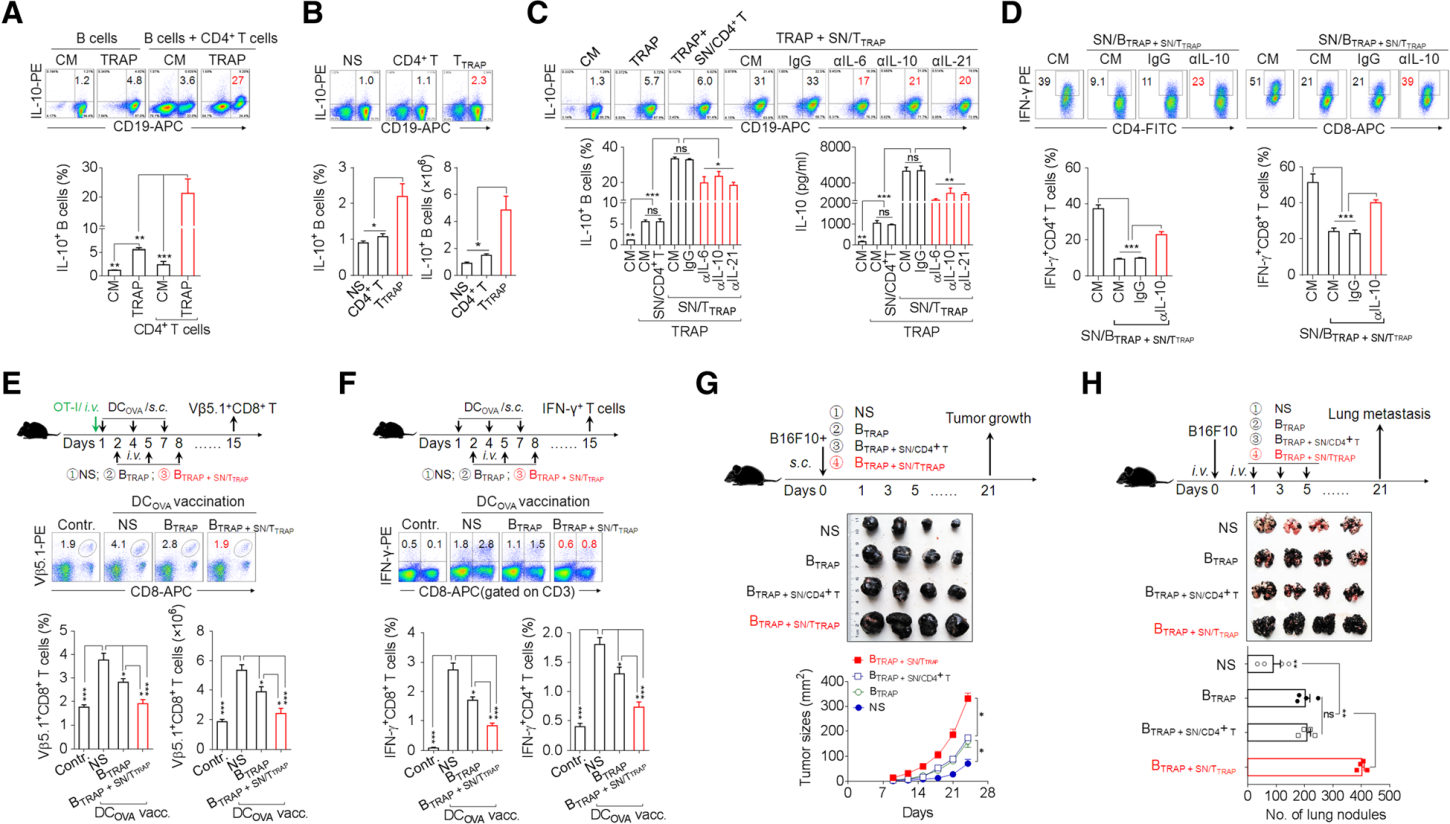
T_TRAP_ enhance Breg differentiation and function via IL-6, IL-10, and IL-21. **a** Flow cytometric assessment of IL-10 expression by splenic B cells after 3 d of co-culture with 3 μg/ml TRAPs or 3 μg/ml TRAPs and an equal number of CD4^+^ T cells. **b** T_TRAP_ were adoptively (i.v.) transferred into C57BL/6 mice (*n* = 3 per group) every other day for 3 times. The frequency and number of splenic IL-10^+^ Bregs 7 days after the last transfer of T_TRAP_ was determined by flow cytometry. **c** SN/T_TRAP_ was pretreated with an anti-IL-6, −IL-10 or -IL-21 neutralizing antibody and co-cultured with splenic B cells and TRAPs for 72 h. The numbers of IL-10^+^ Bregs and IL-10 secretion were determined by flow cytometry and ELISA, respectively. **d** The supernatants from B cells stimulated with 3 μg/ml TRAPs and SN/T_TRAP_ (SN/B_TRAP + SN/TTRAP_) were untreated or pre-treated with an anti-IL-10 neutralizing antibody and then used to culture anti-CD3/28-activated CD4^+^ or CD8^+^ T cells for 3 days. The percentage of IFN-γ^+^ T cells was determined by flow cytometry. **e** C57BL/6 mice were i.v. injected with OT-I splenocytes and vaccinated s.c. with DC_OVA_ on day 1, 4 and 7, following adoptive transfer of B cells induced by TRAPs (B_TRAP_) or by TRAPs and SN/T_TRAP_ (B_TRAP + SN/TTRAP_) on day 2, 5 and 8. On day 15, the frequency and the number of Vβ5.1^+^CD8^+^ T cells in spleens were analyzed by flow cytometry. **f** C57BL/6 mice were vaccinated with DC_OVA_ and transferred with B_TRAP_ or B_TRAP + SN/TTRAP_. At day 15, the frequencies of splenic IFN-γ^+^ CD4^+^ and CD8^+^ T cells were determined after ex vivo re-stimulation. **g, h** B16F10 tumor cells were mixed with B_TRAP_, B_TRAP + SN/CD4+ T_, or B_TRAP + SN/TTRAP_ and injected s.c. into C57BL/6 mice (*n* = 4 per group). The growth of tumor was monitored (g). B16F10 tumor cells were injected i.v. into C57BL/6 mice (*n* = 4 per group) to establish a lung metastasis model. Subsequently, the above-prepared B cells were i.v. transferred every other day for 3 times. Three weeks later, tumor nodules in the lungs were examined (h). Data (mean ± SEM) represent 3 independent experiments. *, *P* < 0.05; **, *P* < 0.01, ***, *P* < 0.001; ns, not significant

To better define the immunosuppressive capacity of T_TRAP_, we examined the impact of T_TRAP_ on regulatory B cell (Breg) differentiation. In accordance with our earlier findings [18], TRAPs induced B cell differentiation into IL-10-producing Bregs (Fig. 6a). Moreover, co-culture of B cells and CD4^+^ T cells in the presence of TRAPs led to a marked increase in Bregs differentiation (Fig. 6a). Consistently, SN/T_TRAP_ could directly promote IL-10^+^ Bregs differentiation and IL-10 secretion (Additional file 2: Figure S6a, b). Next, adoptive transfer of T_TRAP_, but not control CD4^+^ T cells, also significantly increased the frequency and number of IL-10^+^ Bregs in vivo (Fig. 6b). Therefore, TRAPs can promote IL-10^+^ Breg differentiation directly by activating on B cells and indirectly by conditioning CD4^+^ T cells.

We then investigated the mechanism by which T_TRAP_ promote IL-10^+^ Bregs differentiation. In agreement with the above results, culturing B cells in SN/T_TRAP_ together with TRAPs resulted in a synergistic increase the frequencies of IL-10^+^ Bregs and IL-10 secretion as compared to TRAPs or SN/T_TRAP_ alone, whereas the supernatant of control CD4^+^ T cells did not have this effect (Fig. 6c). Neutralizing IL-6, IL-10 or IL-21 partially abolished the effect of SN/T_TRAP_ in promoting IL-10 production of TRAPs-induced B cells (Fig. 6c). These data indicate that secreted cytokines, including IL-6, IL-10, and IL-21, from T_TRAP_ were involved in promoting Bregs differentiation.

Subsequently, the potential regulatory effect of B cells pretreated by TRAPs and SN/T_TRAP_ (B_TRAP + SN/TTRAP_) on the antitumor effector function of T cells was assessed. IFN-γ production by activated CD4^+^ and CD8^+^ T cells was strongly suppressed when these cells were cultured in the supernatants from B_TRAP + SN/TTRAP_ (SN/B_TRAP + SN/TTRAP_), and the suppressive activity of the SN/B_TRAP + SN/TTRAP_ on IFN-γ production by T cell was largely abolished using an anti-IL-10 neutralizing antibody (Fig. 6d). To further investigate the suppressive effects of B_TRAP + SN/TTRAP_ on effector T cell response in vivo, C57BL/6 mice, with or without adoptive transfer of OT-I cells were vaccinated with DC_OVA_ and subsequently were adoptively transferred with B_TRAP + SN/TTRAP_, or B_TRAP_. DC_OVA_ vaccination induced the expansion of Vβ5.1^+^CD8^+^ OT-I T cells in the recipient mice. Adoptive transfer of B_TRAP_ inhibited the expansion of OT-I T cells, and the transfer of B_TRAP + SN/TTRAP_ resulted in a more pronounced and almost complete inhibition of the expansion of OT-I T cells (Fig. 6e). Moreover, adoptive transfer of B_TRAP + SN/TTRAP_ decreased the numbers of IFN-γ^+^ CD8^+^ and CD4^+^ T cells induced by DC_OVA_ vaccination (Fig. 6f) and promoted the growth of B16F10 melanoma cells and their metastasis to the lung (Fig. 6g, h). Taken together, these results suggest that IL-6, IL-10, and IL-21 from T_TRAP_ augment the differentiation and immunosuppressive function of TRAPs-induced B cells to facilitate tumor growth and metastasis.

### Inhibition of autophagosomes formation or IL-6 secretion delay tumor growth

**Fig. 7:**
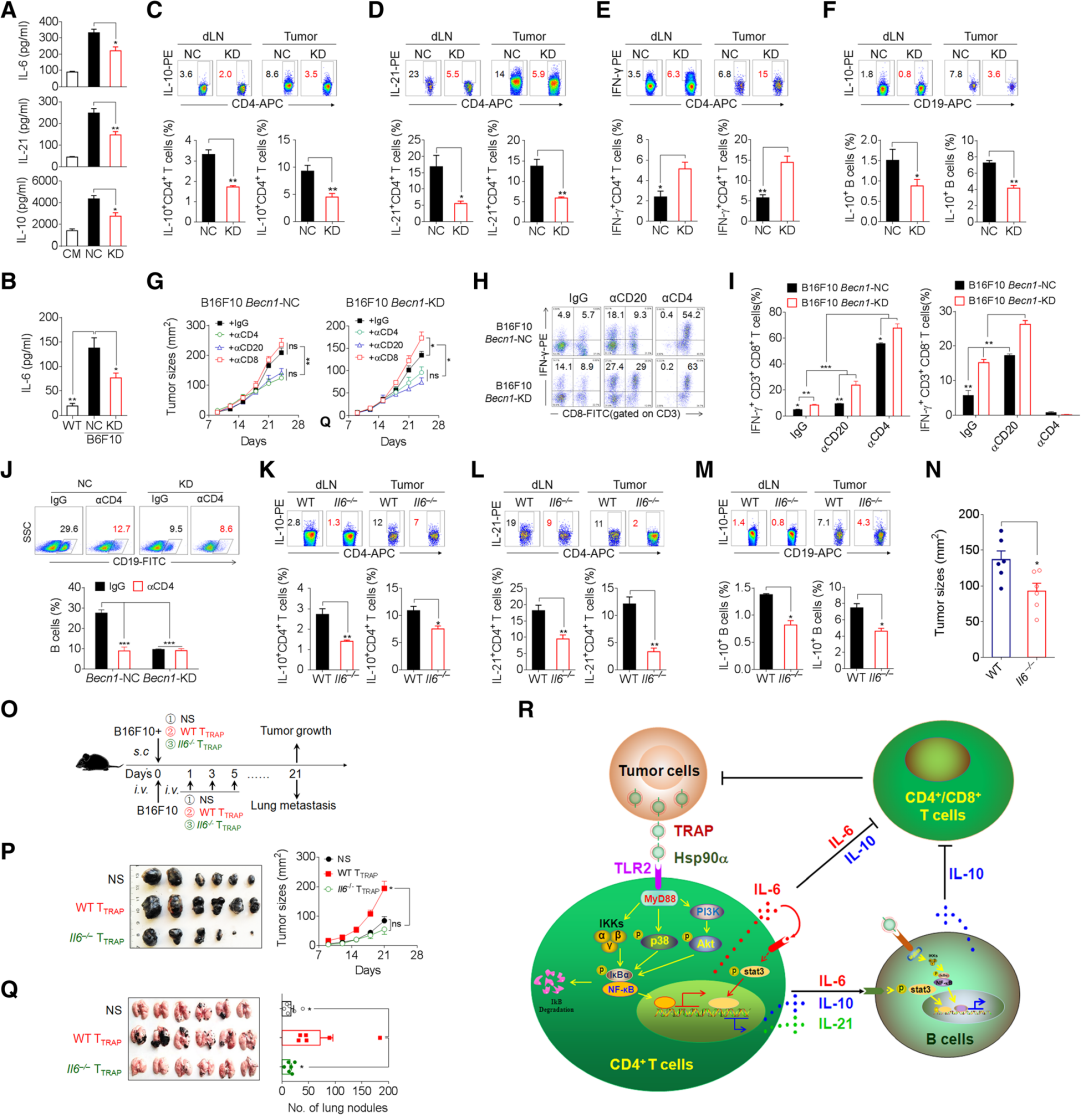
Knockdown of Beclin1 or knockout of IL-6 inhibits tumor growth and alters CD4^+^T and B cells polarization. **a** ELISA of IL-6, IL-10, and IL-21 secretion by CD4^+^ T cells treated with CM, or media from negative control (NC) or *Becn1*-knockdown (KD) B16F10 cells in the presence of anti-CD3/CD28 for 72 h. **b-f** Control (NC) or *Becn1*-KD B16F10 cells were inoculated s.c. into C57BL/6 mice (*n* = 6 per group). Twenty-one days later, serum IL-6 level was measured by ELISA (**b**). The frequency of IL-10^+^ CD4^+^ T cells (**c**), IL-21^+^ CD4^+^ T cells (**d**), IFN-γ^+^ CD4^+^ T cells (**e**), and IL-10^+^ B cells (**f**) in tumor-draining lymph nodes (dLN) or tumor tissues (Tumor) were analyzed by flow cytometry. **g-j** The tumor-bearing mice (*n* = 5 per group) were depleted of either CD4^+^ or CD8^+^ T cells or CD20^+^ B cells by intravenous administration of 250 μg/mouse of anti-mouse CD4 or anti-mouse CD8 antibodies twice weekly throughout the course of tumor growth, or 250 μg of anti-mouse CD20 antibodies, respectively. Control mice were treated with isotype-matched control antibodies. **g** Tumor growth was monitored by calipers. **h, i** The frequency of CD4^+^ IFN-γ^+^ and CD8^+^ IFN-γ^+^ T cells in tumor infiltrating lymphocytes were determined by intracellular staining. **j** The frequency of B cells in tumor infiltrating lymphocytes were determined by flow cytometry. **k-n** WT or *Il6*^−/−^ C57BL/6 mice were inoculated s.c. with B16F10 cells. On day 21, the frequencies of IL-10^+^ CD4^+^ T cells (k), IL-21^+^ CD4^+^ T cells (**l**), and IL-10^+^ B cells (m) in dLN and tumor tissues were evaluated by flow cytometry. **n** Tumor size was measured by caliper. **o, p** B16F10 tumor cells were mixed with WT T_TRAP_ or *Il6*^−/−^ T_TRAP_ and injected s.c. into C57BL/6 mice (*n* = 6 per group). The growth of the tumor was monitored. **o, q** B16F10 tumor cells were i.v. injected into C57BL/6 mice (*n* = 6 per group). Subsequently, WT T_TRAP_ or *Il6*^−/−^ T_TRAP_ were transferred i.v. every other day for 3 times. Three weeks later, tumor nodules in the lungs were examined. **r** A proposed model for the mechanisms and immunosuppressive functions of TRAPs-induced CD4^+^ T cells. Data (mean ± SEM) represent 3 independent experiments. *, *P* < 0.05; **, *P* < 0.01; ***, *P* < 0.001; ns, not significant, by 1-way ANOVA with the Tukey-Kramer multiple test, 2-tailed unpaired t-test or Mann-Whitney U test

Having shown a critical role of TRAPs in the inhibition of anti-tumor immunity, we explored whether inhibition of TRAPs formation by targeting *Becn1*, a gene essential for autophagosome formation, could abolish the generation of tumor-promoting T_TRAP_ (Additional file 2: Figure S7a, b). *Becn1* knock-down in B16F10 cells diminished intracellular LC3-II accumulation and markedly reduced TRAPs secretion (Additional file 2: Figure S7b, c). The culture media collected from *Becn1* knock-down B16F10 cells had reduced ability to induce IL-6, IL-10, and IL-21 in CD4^+^ T cells (Fig. 7a). In the mice bearing knock-down B16F10 tumors, the frequency of IL-21^+^ and IL-10^+^ CD4^+^ T cells in the tumor draining lymph node and tumor tissue and the serum IL-6 level were significantly reduced as compared to those in the mice bearing control tumors (Fig. 7b–d). Moreover, the frequency of IL-10^+^ B cells and IFN-γ^+^CD4^+^ T cells in mice bearing *Becn1* knock-down tumors was markedly decreased and increased, respectively (Fig. 7e, f). Additionally, *Becn1* knock-down B16F10 cells exhibited significantly slower growth in vivo (Additional file 2: Figure S7d). These results indicate that inhibition of tumor autophagosome formation and release could enhance anti-tumor immunity and inhibit tumor growth in vivo.

Furthermore, the growth of both the negative control and *Becn1* knock-down B16F10 tumors was inhibited in mice depleted of B cells or CD4^+^ T cells (Fig. 7g, Additional file 2: Figure S8). Depletion of CD8^+^ T cells resulted in accelerated growth of *Becn1* knock-down but not negative control tumors (Fig. 7g, Additional file 2: Figure S8). Besides, the frequency of IFN-γ-producing CD4^+^ T cells and CD8^+^ T cells in *Becn1* knock-down tumor tissue was markedly increased (Fig. 7h, i). Notably, B-cell or CD4^+^ T-cell depletion resulted in a significant increase of the percentage of intra-tumoral IFN-γ^+^ CD4^+^ or CD8^+^ T cells (Fig. 7h, i). The frequency of tumor-infiltrating B cells was markedly reduced upon CD4^+^ T cell depletion (Fig. 7j). These results suggest that the effector function of CD8^+^ T cells in the tumors was dampened by CD4^+^ T cells or B cells. In conclusion, TRAPs-educated CD4^+^ T cells play an important role in promoting tumor growth by inhibiting effector T cell function.

To determine the role of CD4^+^ T cell-derived IL-6 in the differentiation of IL-10- and IL-21-producing CD4^+^ T cells and IL-10-producing Bregs in vivo, WT or *Il6*^−/−^ mice were s.c. inoculated with B16F10 cells. Consistent with previous results, the frequencies of IL-10^+^ and IL-21^+^ CD4^+^ T cells (Fig. 7k, l) and IL-10^+^ B cells (Fig. 7m) in tumor-draining lymph nodes and tumor tissues from *Il6*^−/−^ tumor-bearing mice were significantly decreased. Accordingly, B16F10 tumors grew more slowly in *Il6*^−/−^ mice than in WT mice (Fig. 7n). We then inoculated mice with B16F10 cells together with either WT T_TRAP_ or *Il6*^−/−^ T_TRAP_. Mice co-inoculated with B16F10 cells and WT T_TRAP_ showed accelerated growth and lung metastasis as compared to those inoculated with B16F10 cells alone (Fig. 7o-q). In contrast, co-inoculation of B16F10 cells with *Il6*^−/−^ T_TRAP_ resulted in no enhancement of tumor growth and lung metastasis, and the mice even exhibited slightly, albeit not statistically significant, retarded tumor growth (Fig. 7o-q). These results corroborate the conclusion that T_TRAP_ rely on IL-6 to dampen T cell-mediated antitumor immunity and foster tumor progression, and suggest that targeting TRAPs or IL-6 may be an effective therapeutic strategy for improving cancer immunotherapy.

## Discussion

In addition to soluble factors, tumor cell-derived extracellular vesicles are being recognized as critical modulators of host anti-tumor immunity during tumor progression [7, 8, 18, 19, 24]. Among them are autophagosomes generated by secretory autophagy. In contrast to canonical autophagy that functions in a primarily degradative capacity to sustain cellular metabolism and homeostasis and is often induced conditions of cellular stress, such as nutrient starvation, organelle damage, and pathogen infection, secretory autophagy is a non-degradative mechanism for cellular trafficking and unconventional protein secretion [10, 11, 13, 14, 25]. Secretory autophagosomes fail to fuse with lysosomes, but are released into the extracellular environment through fusing with the plasma membrane or other pathways [15, 26]. Abundant autophagosomes have been detected in gastrointestinal tumors and invasive melanomas and have been associated with tumor cell proliferation, metastasis, and poor prognosis [27, 28]. Our previous studies showed that extracellular autophagosomes harvested from the supernatant of tumor cells or malignant effusions and ascites of cancer patients, which we have termed as TRAPs, could promote the generation of IL-10^+^ Bregs, reactive oxygen species (ROS)-producing neutrophils, and PD-L1^hi^ macrophages exerting immunoinhibitory activities [18–20].

CD4^+^ T cells that infiltrate advanced solid tumors consist of different effector cells, such as Th1, Th2, Th17, Tfh or regulatory T cells (Tregs), with distinct impact on anti-tumor immunity, immune escape, angiogenesis and metastasis [2, 4, 29], but the influence of the tumors on CD4^+^ effector T cell differentiation remains incompletely understood. Here, we have revealed a TRAPs-mediated regulatory mechanism of CD4^+^ T cells differentiation whereby HSP90α on the surface of TRAPs educate CD4^+^ T cells via a TLR2–autocrine IL-6 cascade to express IL-10 and IL-21 and engender immune suppression to promote tumor growth and metastasis (Fig. 7r). Our findings have revealed TRAPs as one of the tumor-derived extracellular vesicles that could inhibit anti-tumor immune response by enhancing the generation of immunosuppressive cells.

TLRs play crucial roles in the innate host defense as well as the control of adaptive immunity [30, 31]. Our findings indicated TLR2 as a key receptor for TRAPs-mediated IL-6 expression by CD4^+^ T cells. Exogenous pathogen-associated molecular patterns (PAMPs) and endogenous DAMPs can be recognized by TLRs to trigger the production of various inflammatory mediators [30]. The current findings showed that TRAPs-mediated regulation of CD4^+^ T cell differentiation involved membrane-associated Hsp90α. Evidences suggested that extracellular Hsp90α could be released to the extracellular space via unconventional secretion, such as exosomes and necrosis [32]. We observed Hsp90α on the surface of TRAPs, indicating that secretory autophagosomes may also be involved in the release of Hsp90α. Moreover, extracellular Hsp90α was reported to function as a DAMP and provoke biological effects through cell surface receptors, including TLRs and CD91 [23, 33]. Early work showed that heat shock proteins gp96, Hsp90, Hsp70, and calreticulin could function as potential adjuvants to stimulate DC antigen cross-presentation and maturation through the CD91 receptor [33], but Hsp90α was more recently found to also stimulate tumor proliferation and metastasis through binding to cancer cell surface CD91 and be positively correlated with tumor malignancy in cancer patients [34–36]. The present study uncovers a new role of Hsp90α on the surface of TRAPs as a cancer-associated pathological factor that interferes with host anti-tumor immunity.

Chronic inflammation and increased levels of inflammatory mediators at the tumor site can reroute the immunomodulatory response towards a cancer-promoting direction [4, 37, 38]. IL-6 has a profound effect on CD4^+^ T cells survival and proliferation [39]. Otherwise, studies also showed that IL-6 has inhibitory effects via the induction of IL-10-producing T and B cells [40, 41]. Moreover, IL-6 also dampens Th1 differentiation and inhibits CD8^+^ T cell activation and cytokine production [42, 43]. Consistent with the above results, we provided evidences that TRAPs stimulated IL-10 and IL-21 production in CD4^+^ T cells via an autocrine IL-6 loop. Moreover, IL-6 from T_TRAP_ remarkably suppressed T cell anti-tumor effector function. IL-21 has been identified to be derived mainly from Tfh cells, which was thought to regulate the proliferation, class switching, and plasmacytoid differentiation of B cells and promote the generation and proliferation of human antigen-specific cytotoxic T-cell responses [4, 44, 45]. Mounting evidences have shown that IL-21 also has anti-inflammatory activities by inhibiting DC maturation and stimulating IL-10 production in T and B cells [46–48]. Nonetheless, the role of CD4^+^ T cells in Bregs differentiation in the tumor microenvironment has not been addressed. In our investigation, the IL-21^+^ T_TRAP_ displayed Tfh-associated molecules CXCR5 and Bcl-6. Interestingly, IL-6, IL-10, and IL-21 secretion by T_TRAP_ synergistically enhanced TRAPs-elicited Breg differentiation and immunosuppressive function. These findings together imply that T_TRAP_-derived IL-21 is a pleiotropic effector that can either facilitate or thwart tumor growth depending on the cytokine milieu in the tumor microenvironment, warranting careful consideration of the selective targeting of IL-6 or IL-21 for the treatment of cancer in the future.

Many recent studies have suggested that inhibiting tumor autophagy may have anti-tumor effects by modulating the tumor microenvironment [49–51]. Consistent with this notion, we found that inhibiting autophagy by targeting the key autophagy gene *Becn1*, which led to a substantial decrease in extracellular TRAPs, could inhibit tumor growth in mice. Of note, inhibiting autophagy resulted in a significant decrease in the frequency of IL-10^+^ B cells, IL-21^+^ and IL-10^+^CD4^+^ T cells, as well as a significant increase in IFN-γ^+^CD4^+^ T cells, in the tumor-draining lymph nodes and tumor tissue. Thus, intervening tumor release of TRAPs could be an effective strategy for cancer therapy.

## Conclusions

In this study, we have revealed that TRAPs can educate CD4^+^ T cells to promote tumor growth and metastasis through an HSP90α–TLR2–IL-6–IL-10/IL-21 axis and the induction of IL-10^+^ Bregs. Our study reveals a novel cellular and molecular mechanism of how tumor-derived extracellular vesicles regulate CD4^+^ effector T cell function and highlights TRAPs and their membrane-bound DAMPs as important therapeutic targets to reverse the immunosuppressive tumor microenvironment.

## Additional files

Additional file 1: **Table S1.** Clinical and demographic characteristics of the patients presenting with malignant pleural effusions or ascites. **Table S2**. Antibodies used in flow cytometry. **Table S3**. Primer sequences for qPCR used in our study. **Table S4**. Antibodies used for immunoblotting. (DOCX 25 kb)

Additional file 2: **Figure S1.** Effect of TRAPs on differentiation of CD4^+^ T cells. **Figure S2**. Comparison of LC3B^+^ EVs and LC3B^−^ EVs in instructing CD4^+^ T cells. **Figure S3** TRAPs induce Akt/p38/NF-κB/STAT3 activation in CD4^+^ T cells via the TLR2–MyD88 pathway. **Figure S4** IL-10 and IL-21 production by TRAPs-induced CD4^+^ T cells occurs via autocrine IL-6. **Figure S5** Hsp90α on TRAPs is essential for the induction of IL-6 secretion from CD4^+^ T cells. **Figure S6.** The secretion of cytokines from TRAPs-induced CD4^+^ T cells (T_TRAP_) mediate IL-10-producing B cell differentiation. **Figure S7** Evaluation of Beclin-1 expression in B16F10 cells. **Figure S8** Depletion of selected cellular subsets during tumor growth. (DOCX 2.11 mb)

## Data Availability

The datasets analyzed during the current study are available from the corresponding author on reasonable request.

## Abbreviations

APCs: Antigen-presenting cells
Bregs: Regulatory B cells
CFSE: Carboxyfluorescein succinimidyl ester
DAMPs: Damage-associated molecular pattern molecules
dLNs: Draining lymph nodes
EVs: Extracellular vesicles
HMGB1: High mobility group box 1
HSP: Heat shock protein
KD: Knock down
KO: Knock out
mAb: Monoclonal antibody
NS: Normal saline
PAMPs: Pathogen-associated molecular patterns
PBMC: Peripheral blood mononuclear cell
ROS: Reactive oxygen species
TB: Tumor-bearing
TF: Tumor-free
TLRs: Toll-like receptors
TRAPs: Tumor cell-released autophagosomes
WT: Wild-type
