# Supplementary material for: Tumor-released autophagosomes induces CD4+ T cell-mediated immunosuppression via a TLR2–IL-6 cascade
Source: J Immunother Cancer. 2019 Jul 12;7:178. doi: 10.1186/s40425-019-0646-5 (PMC6625067; doi:10.1186/s40425-019-0646-5)
Supplement: Supplementary file 1 — Table S1. Clinical and demographic characteristics of the patients presenting with malignant pleural effusions or ascites. Table S2. Antibodies used in flow cytometry. Table S3. Primer sequences for qPCR used in our study. Table S4. Antibodies used for immunoblotting. (DOCX 25 kb) [file 40425_2019_646_MOESM1_ESM.docx]

**Table. S1 Clinical and demographic characteristics of the patients presenting with malignant pleural effusions or ascites**

| **Patient ID** | **Age/Sex** | **Primary**  **tumor site** | **Histologic diagnosis** | **Pleural effusions/ascites** | **Stage** |
| --- | --- | --- | --- | --- | --- |
| 01 | 55/F | Ovary | Adenocarcinoma | Ascites | IV |
| 02 | 54/M | Lung | Adenocarcinoma | Pleural effusions | IV |
| 03 | 63/M | Stomach | Adenocarcinoma | Pleural effusions | IV |
| 04 | 70/M | Esophagus | Adenocarcinoma | Pleural effusions | IV |
| 05 | 55/F | Gallbladder | Adenocarcinoma | Ascites | IV |
| 06 | 64/M | Colon | \| Colorectal adenocarcinoma \| \| --- \| | Ascites | IV |
| 07 | 49/F | Liver | \| Hepatocellular carcinoma \| \| --- \| | Ascites | IV |
| 08 | 65/M | Pancreas | Adenocarcinoma | Ascites | IV |

**Table. S2**

**Table S2. Antibodies used in flow cytometry**

| **Antibody** | **Clone** | **Supplier** |
| --- | --- | --- |
| APC Rat Anti-Mouse CD4 | RM4-5 | BD Biosciences |
| FITC Rat Anti-Mouse CD4 | GK1.5 | Biolegend |
| APC Rat Anti-Mouse CD8 | 53-6.7 | eBioscience |
| FITC Rat Anti-Mouse CD8 | 53-6.7 | Biolegend |
| PE Rat Anti-Mouse IL-21 | FFA21 | eBioscience |
| PE Rat Anti-Mouse CXCR5 | L138D7 | Biolegend |
| FITC Rat Anti-Mouse ICOS | C398.4A | Biolegend |
| APC Rat Anti-Mouse CD19 | 6D5 | Biolegend |
| PE Rat Anti-Mouse IL-10 | JES5-16E3 | Biolegend |
| APC Rat Anti-Mouse IL-10 | JES5-16E3 | Biolegend |
| PE Rat Anti-Mouse IFN-γ | XMG1.2 | BD Biosciences |
| PE Rat Anti-Mouse IL-4 | 11B11 | BD Biosciences |
| PE Rat Anti-Mouse IL-6 | MP5-20F3 | eBioscience |
| PE Rat Anti-Mouse IL-17A | eBio17B7 | eBioscience |
| PE Rat Anti-Mouse Bcl-6 | 7D1 | Biolegend |
| PE-Cy5.5 Rat Anti-Mouse Foxp3 | FJK-16s | eBioscience |
| PE Rat Anti-Mouse TLR2 | CB225 | Biolegend |

**Table S3. Primer sequences for qPCR used in our study**

| **Gene** | **Sequence** | |
| --- | --- | --- |
| Mouse *Foxp3* | Forward | 5’-CCTTCTCGCTCTCCACTC-3’ |
|  | Reverse | 5’-CACCTATGCCACCCTTATC-3’ |
| Mouse *Il17a* | Forward | 5’-GTTGACCTTCACATTCTGG-3’ |
|  | Reverse | 5’-TGTCTCTGATGCTGTTGCT-3’ |
| Mouse *Ifng* | Forward | 5’-ACTCCTTTTCCGCTTCCT-3’ |
|  | Reverse | 5’-CACACCTGATTACTACCTTCTTC-3’ |
| Mouse *Il4* | Forward | 5’-CTCTCTGTGGTGTTCTTCGT-3’ |
|  | Reverse | 5’-TCATCCTGCTCTTCTTTCTC-3’ |
| Mouse *Il10* | Forward | 5’-CGCAGCTCTAGGAGCATGTG-3’ |
|  | Reverse | 5’-GCTCTTACTGACTGGCATGAG-3’ |
| Mouse *Tgfb1* | Forward | 5’-GAGCCCGAAGCGGACTACTA-3’ |
|  | Reverse | 5’-TGGTTTTCTCATAGATGGCGTTG-3’ |
| Mouse *Il6* | Forward | 5’-ACAAAGCCAGAGTCCTTCAGAGAG-3’ |
|  | Reverse | 5’-TTGGATGGTCTTGGTCCTTAGCCA-3’ |
| Mouse *Il2* | Forward | 5’-CCTTGCTAATCACTCCTCAC-3’ |
|  | Reverse | 5’-CTGTGCTTCCGCTGTAGA-3’ |
| Mouse *Il17f* | Forward | 5’-AGCCAACTTTTAGGAGCA-3’ |
|  | Reverse | 5’-GTCAGGAAGACAGCACCA-3’ |
| Mouse *Tnf* | Forward | 5’-ACGGCATGGATCTCAAAGAC-3’ |
|  | Reverse | 5’-AGATAGCAAATCGGCTGACG-3’ |
| Mouse *Il22* | Forward | 5’-AAGGTGCGGTTGACGATGAT-3’ |
|  | Reverse | 5’-AACTGTTGACACTTGTGCGA-3’ |
| Mouse *Il21* | Forward | 5’-AGGTCCAATGTGTTCCCACC-3’ |
|  | Reverse | 5’-TGCACAGCAGTCTTGAACCT-3’ |
| Mouse *Il8* | Forward | 5’-TTCACCCATGGAGCATCAGG-3’ |
|  | Reverse | 5’-CTAGGCATCTTCGTCCGTCC-3’ |
| Mouse *Il1b* | Forward | 5’-TCTTTGAAGTTGACGGACCC-3’ |
|  | Reverse | 5’-TGAGTGATACTGCCTGCCTG-3’ |
| Mouse *Gapdh* | Forward | 5’-CGAAGGTGGAAGAGTGGGAG-3’ |
|  | Reverse | 5’- TGAAGCAGGCATCTGAGGG-3’ |
| Mouse *Becn1* | Forward | 5’-ACTGGGTTTTGATGGAATAGG-3’ |
|  | Reverse | 5’-TAAGGAGTTGCCGTTATACTGT-3’ |
| Human *IL6* | Forward | 5’- TGCCTCTTTGCTGCTTTCACA-3’ |
|  | Reverse | 5’- TCGGTCCAGTTGCCTTCTCCC-3’ |
| Human *GAPDH* | Forward | 5’- GGACCTGACCTGCCGTCTAG-3’ |
|  | Reverse | 5’- GTAGCCCAGGATGCCCTTGA-3’ |

**Table S4. Antibodies used for immunoblotting**

| **Antibody** | **Clone** | **Dilution** | **Supplier** |
| --- | --- | --- | --- |
| p38 | E229 | 1:1000 | Abcam |
| Phospho-p38 (T180+Y182) | EPR18120 | 1:1000 | Abcam |
| Akt (pan) | C67E7 | 1:1000 | CST |
| Phospho-Akt (Ser473) | D9E | 1:2000 | CST |
| STAT3 | AG0360 | 1:2000 | Proteintech |
| Phospho-STAT3 (Y705) | EP2147Y | 1:3000 | Abcam |
| IKKα | 3G12 | 1:1000 | CST |
| Phospho-IKKα/β (Ser176/180) | 16A6 | 1:1000 | CST |
| IκBα | L35A5 | 1:1000 | CST |
| Phospho-IκBα (Ser32/36) | 5A5 | 1:1000 | CST |
| p65 | D14E12 | 1:1000 | CST |
| Phospho-p65 (Ser536) | 93H1 | 1:1000 | CST |
| Phospho-JNK1/2 (Thr183/Tyr185) | 81E11 | 1:1000 | CST |
| Phospho-ERK1/2 (Thr202/Tyr204) | D13.14.4E | 1:2000 | CST |
| LC-3b | NB100-2220 | 1:1000 | Novus |
| HMGB1 | NB100-2322 | 1:1000 | Novus |
| Hsp70 | 5A5 | 1:1000 | Abcam |
| Hsp60 | 4B9/89 | 1:1000 | Abcam |
| Hsp90α | ADI-SPS-771-D | 1:1000 | Enzo |
| Beclin1 | 11306-1-AP | 1:1000 | Proteintech |
| GAPDH | 1E6D9 | 1:2000 | Proteintech |
| β-Tublin | AG0136 | 1:1000 | Proteintech |
| HRP conjugate-Goat anti-mouse IgG (H+L) | SA00001-1 | 1:2000 | Proteintech |
| HRP conjugate-Goat anti-rabbit IgG (H+L) | SA00001-2 | 1:2000 | Proteintech |
