# Supplementary material for: Tumor-released autophagosomes induces CD4+ T cell-mediated immunosuppression via a TLR2–IL-6 cascade
Source: J Immunother Cancer. 2019 Jul 12;7:178. doi: 10.1186/s40425-019-0646-5 (PMC6625067; doi:10.1186/s40425-019-0646-5)
Supplement: Supplementary file 2 — Figure S1. Effect of TRAPs on differentiation of CD4+ T cells. Figure S2. Comparison of LC3B+ EVs and LC3B− EVs in instructing CD4+ T cells. Figure S3 TRAPs induce Akt/p38/NF-κB/STAT3 activation in CD4+ T cells via the TLR2–MyD88 pathway. Figure S4 IL-10 and IL-21 production by TRAPs-induced CD4+ T cells occurs via autocrine IL-6. Figure S5 Hsp90α on TRAPs is essential for the induction of IL-6 secretion from CD4+ T cells. Figure S6. The secretion of cytokines from TRAPs-induced CD4+ T cells (TTRAP) mediate IL-10-producing B cell differentiation. Figure S7 Evaluation of Beclin-1 expression in B16F10 cells. Figure S8 Depletion of selected cellular subsets during tumor growth. (DOCX 2.11 mb) [file 40425_2019_646_MOESM2_ESM.docx]

**Additional file 2**

**Figure S1**


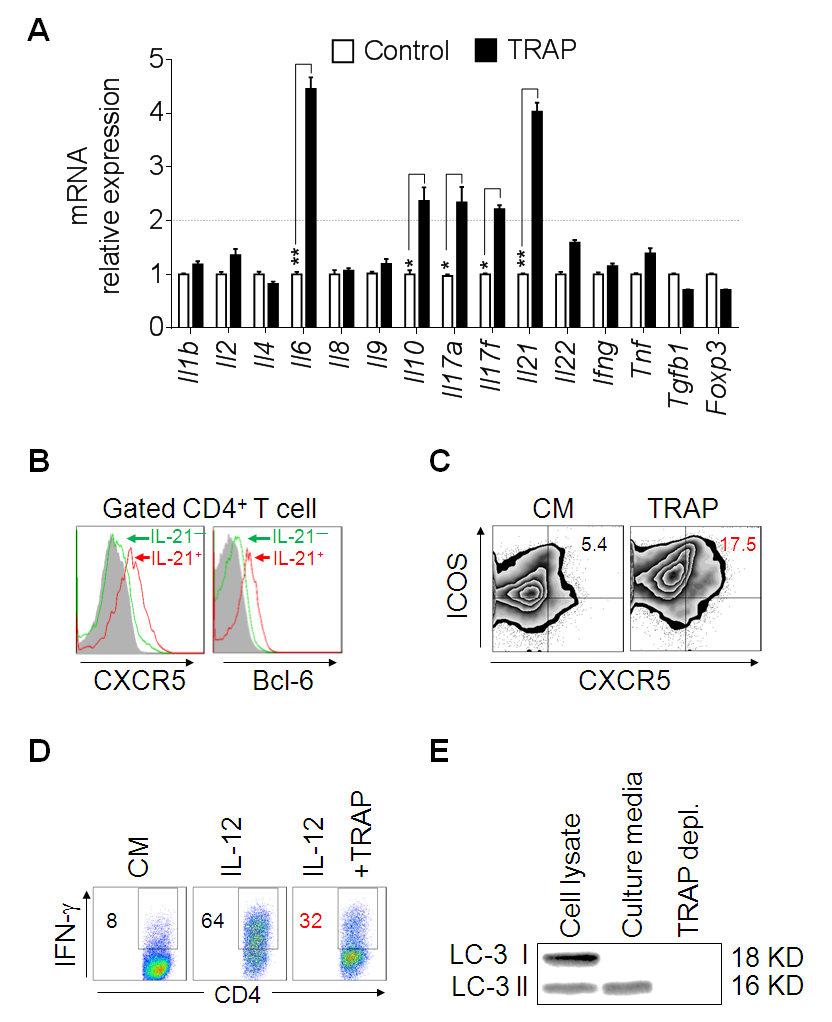


**Figure. S1** Effect of TRAPs on differentiation of CD4^+^ T cells. **a** The purified CD4^+^ T cells were activated by anti-CD3/CD28 mAbs and co-cultured with or without TRAPs (3 μg/ml) for 24 h. The expression of specific T cell cytokines or transcription factor was analyzed by real-time PCR. **b-d** The purified CD4^+^ T cells were activated by anti-CD3/CD28 mAbs and co-cultured with or without TRAPs (3 μg/ml) or IL-12 (5 ng/ml) for 72 h. The expression of BCL-6 and CXCR5 in TRAPs-induced IL-21^+^CD4^+^ T cells, the frequencies of CXCR5^+^ICOS^+^CD4^+^ T cells, and the frequencies of IFN-γ^+^CD4^+^ T cells were assessed by flow cytometry. **e** B16F10 **t**umor cell culture media and TRAP-depleted culture media (TRAPs-depl.) by ultracentrifugation were concentrated and solubilized in lysis buffer. The expressing of autophagosomes specific marker LC-3II was detected by Western blot. *, P < 0.05; **, P < 0.01, by Mann-Whitney U test.

**Figure S2**


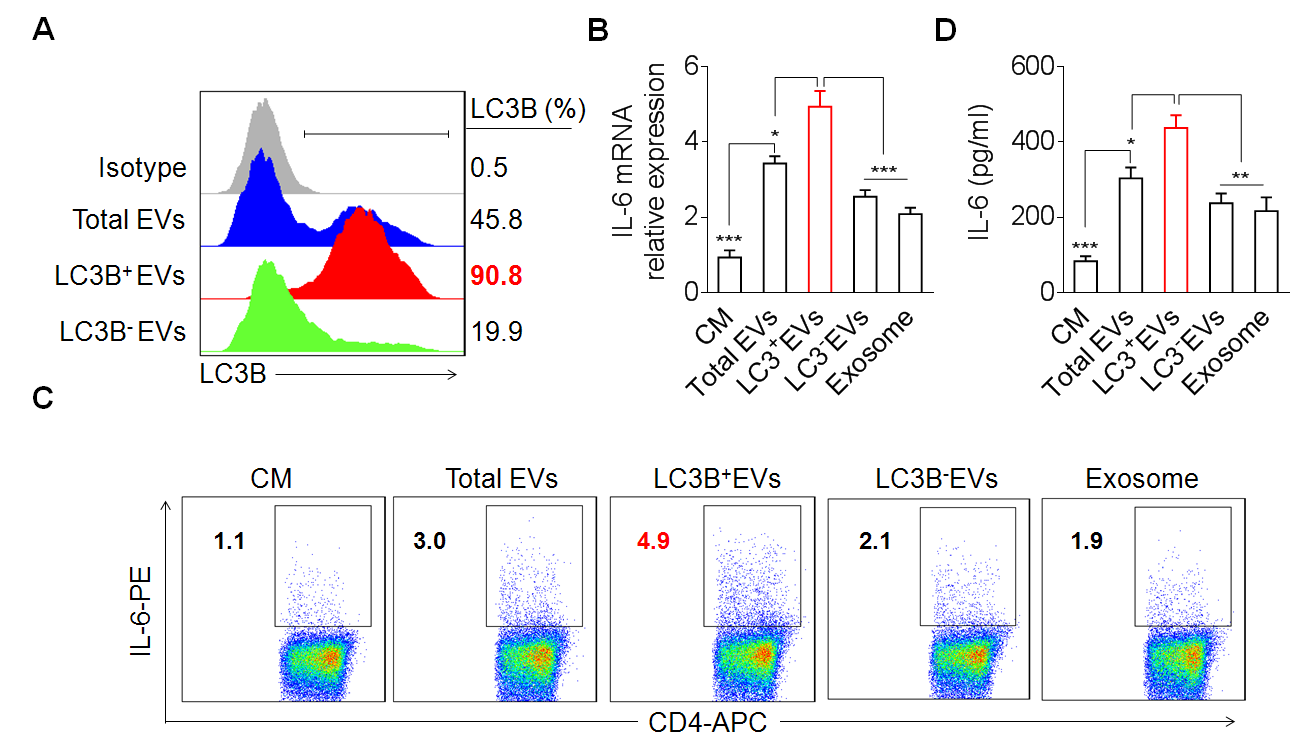


**Figure. S2**. Comparison of LC3B^+^ EVs and LC3B^–^ EVs in instructing CD4^+^ T cells. **a** LC3B^+^ EVs and LC3B^–^ EVs were sorted from total EVs derived from B16F10 culture supernatant, and LC3B expression was determined by flow cytometry. **b, c** Purified CD4^+^ T cells were treated with 3 μg/ml total EVs, LC3B^+^ EVs, LC3B^–^ EVs or exosome for 24 h. The expression of IL-6 was detected by qRT-PCR (**b**) and flow cytometry (**c**). **d** IL-6 production in the supernatant was assessed by ELISA. *, P < 0.05; **, P < 0.01, by Mann-Whitney U test.

**Figure S3**

**
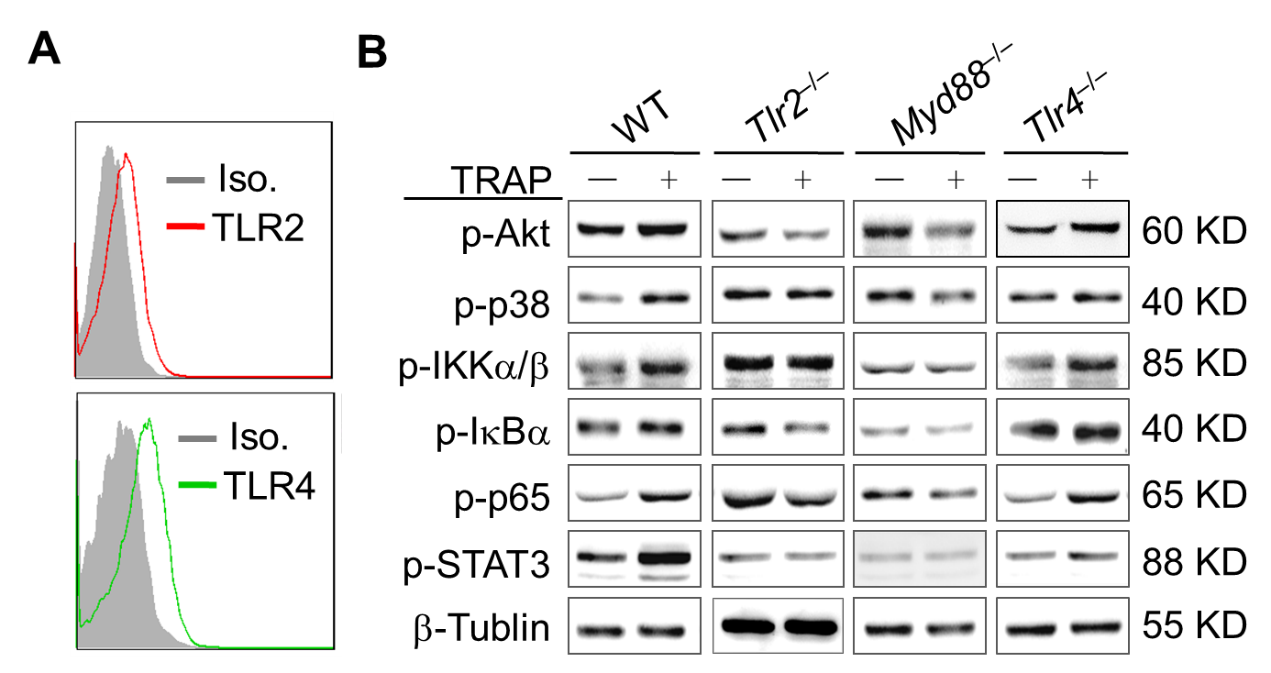
**

**Figure. S3** TRAPs induce Akt/p38/NF-κB/STAT3 activation in CD4^+^ T cells via the TLR2–MyD88 pathway. **a** Flow cytometric determination of the expression of surface TLR2 and TLR4 on CD4^+^ T cells. **b** CD4^+^ T cells purified from WT, *Tlr2*^–/–^, *Tlr4*^–/–^, or *Myd88*^–/–^ mice were stimulated with 3 μg/ml TRAPs for 2 h. The phosphorylation of p38, Akt, IKKα/β, IκBα, p65, and STAT3 were determined by western blot. Results represent 3 separate experiments.

**Figure S4**


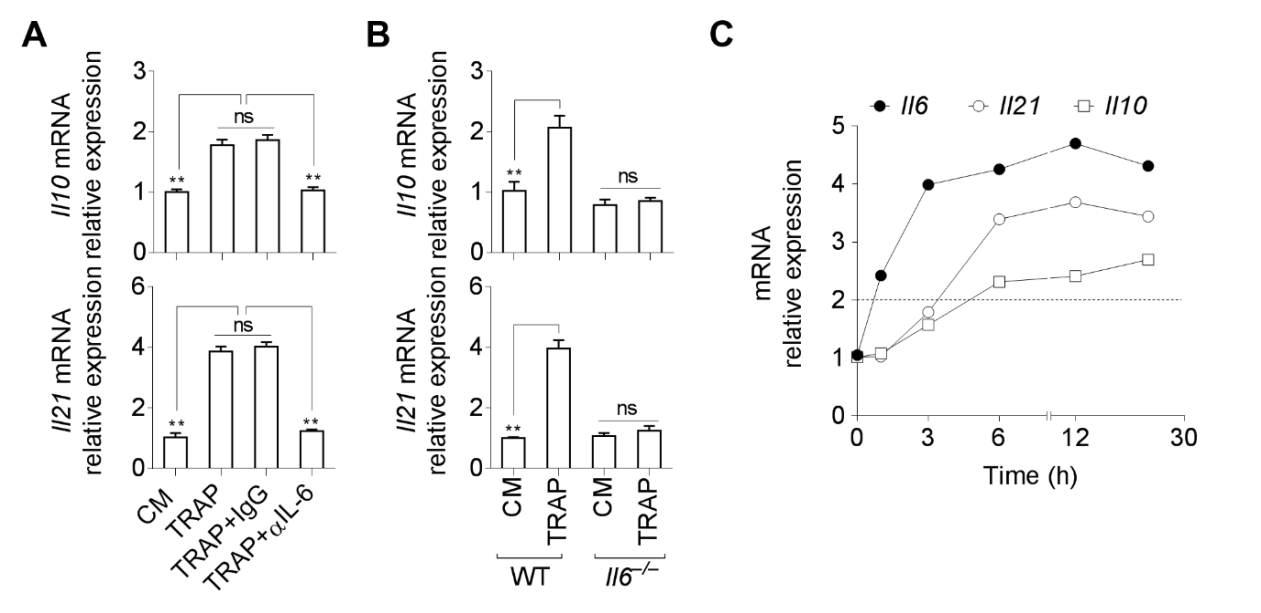


**Figure. S4** IL-10 and IL-21 production by TRAPs-induced CD4^+^ T cells occurs via autocrine IL-6. **a** Splenic CD4^+^ T cells purified from WT C57BL/6 mice were activated with anti-CD3/CD28 mAbs in the presence of TRAPs with or without a neutralizing anti-IL-6 mAb (1 μg/ml) for 24 h. *Il10* and *Il21* expression in CD4^+^ T cells was determined by qRT-PCR. **b** Splenic CD4^+^ T cells purified from wild type or *Il6*^–/–^ C57BL/6 mice were treated with or without TRAPs in the presence of anti-CD3/CD28 mAbs for 24 h. *Il10* and *Il21* expression in CD4^+^ T cells was analysis by qRT-PCR. **c** Purified splenic CD4^+^ T cells were treated with TRAPs for the indicated period in the presence of anti-CD3/CD28 mAbs. *Il6*, *Il10* and *Il21* expression in CD4^+^ T cells was determined by qRT-PCR. Data (mean ± SEM) represent 3 independent experiments. **, P < 0.01, ns, not significant, by Mann-Whitney U test.

**Figure S5**


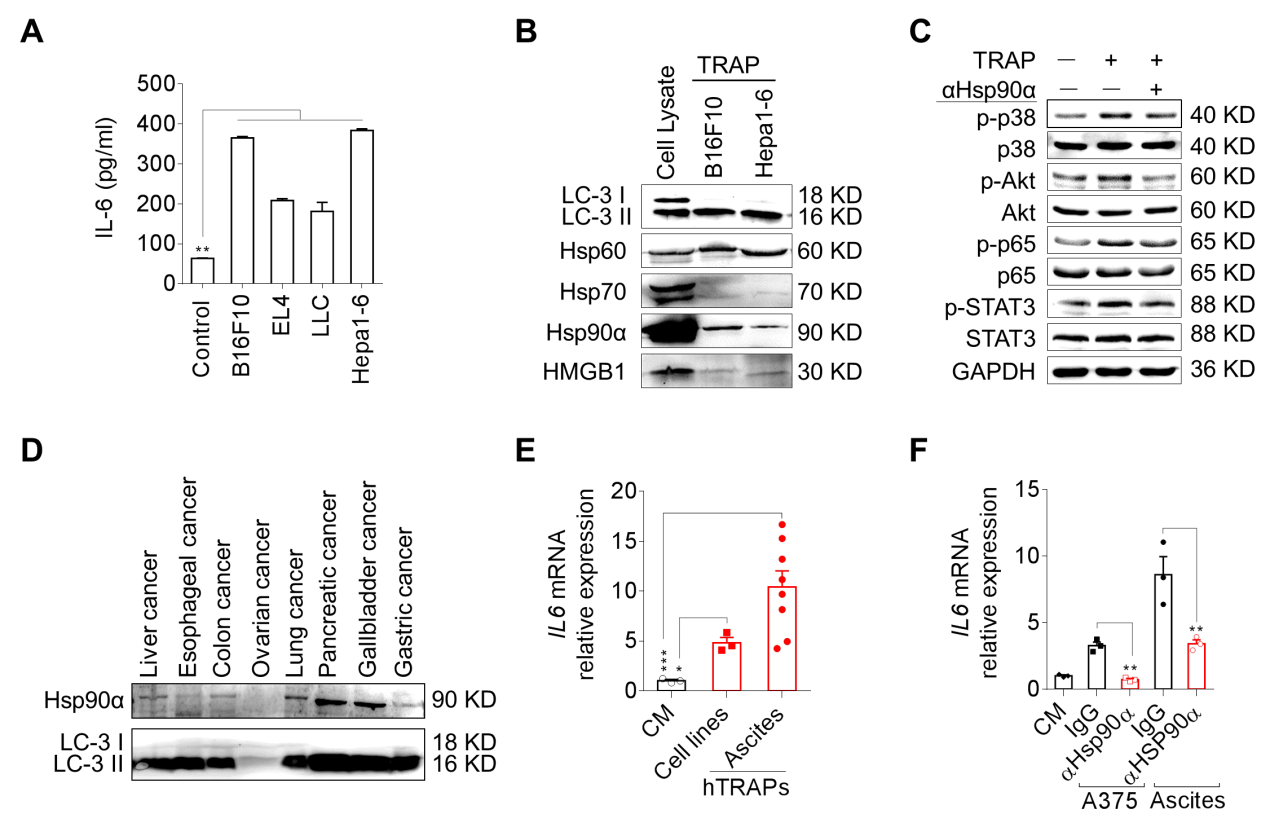


**Figure. S5** Hsp90α on TRAPs is essential for the induction of IL-6 secretion from CD4^+^ T cells. **a** Purified CD4^+^ T cells were activated with anti-CD3/CD28 and co-cultured with TRAPs from mouse B16F10 melanoma, Hepa1-6 hepatic carcinoma, LLC lung carcinoma or EL4 lymphoma cells for 72 h. IL-6 levels in supernatants were measured by ELISA. **b** HSP60, HSP70, HSP90α and HMGB1 expression in TRAPs was determined by western blot. **c** TRAPs were pretreated with an anti-Hsp90α blocking antibody and then co-cultured with CD4^+^ T cells for 2 h**.** The phosphorylation of p38, Akt, p65, and STAT3 was determined by western blot. **d** Hsp90α and LC-3I/II expression in hTRAPs from the effusions and ascites of 8 cancer patients were examined by western blot. **e, f** Purified CD4^+^ T cells from healthy human peripheral blood were activated by anti-CD3/CD28 Abs and co-cultured with 3 μg/ml hTRAPs or hTRAPs pretreated with an 3 μg/ml anti-Hsp90α or control antibody for 24 h. *IL6* expression in CD4^+^ T cells was analysis by qRT-PCR. Data (mean ± SEM) represent 3 independent experiments. *, P < 0.05; **, P < 0.01, ***, P < 0.001.

**Figure S6**

**
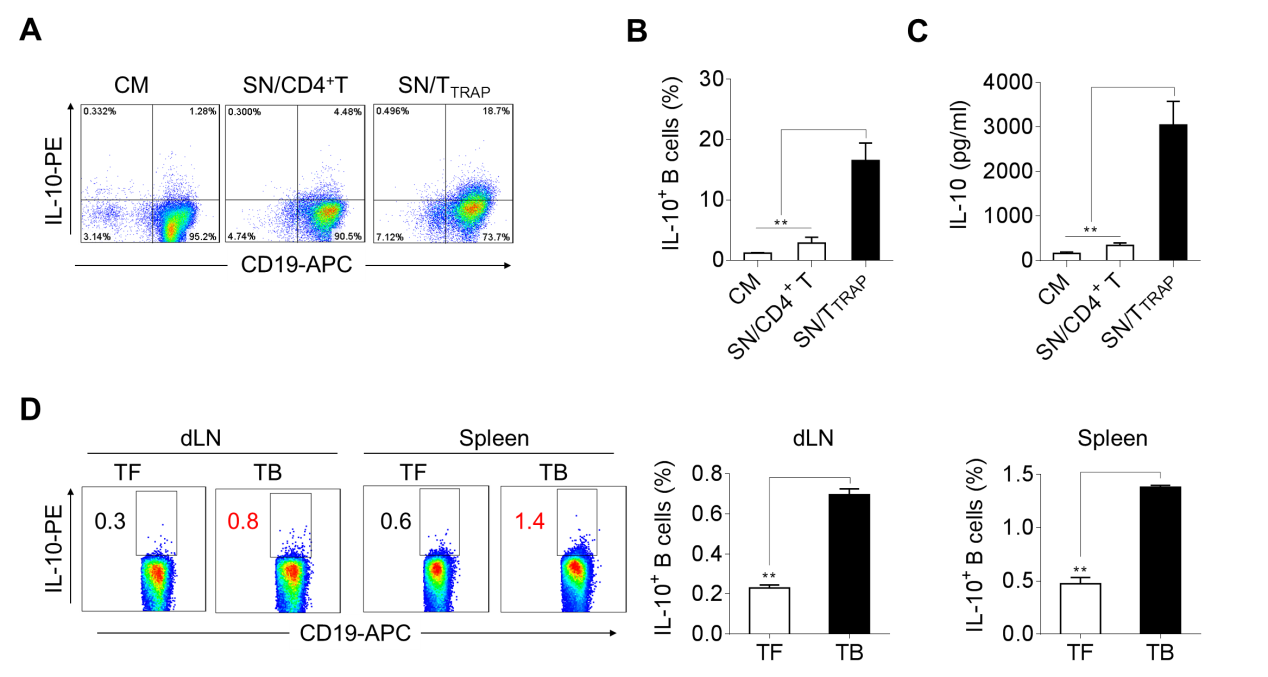
**

**Figure S6.** The secretion of cytokines from TRAPs-induced CD4^+^ T cells (T_TRAP_) mediate IL-10-producing B cell differentiation. **a-c** Purified splenic B cells were co-cultured with supernatants from control CD4^+^ T cells (SN/CD4^+^T) or T_TRAP_ (SN/T_TRAP_) for 72 h and the frequency and secretion of IL-10 were determined by flow cytometry and ELISA. **d** Wild type C57BL/6 mice (*n* = 6 per group) were s.c. inoculated with B16F10 cells. On day 21, the frequencies of IL-10^+^ Bregs in the tumor-draining lymph nodes (dLN) and spleens of tumor-free (TF) or tumor-bearing (TB) mice were evaluated by flow cytometry. Data (mean ± SEM) represent 3 independent experiments. **, P < 0.01, ***, P < 0.001, by 2-tailed t-test or Mann Whitney U test.

**Figure S7**


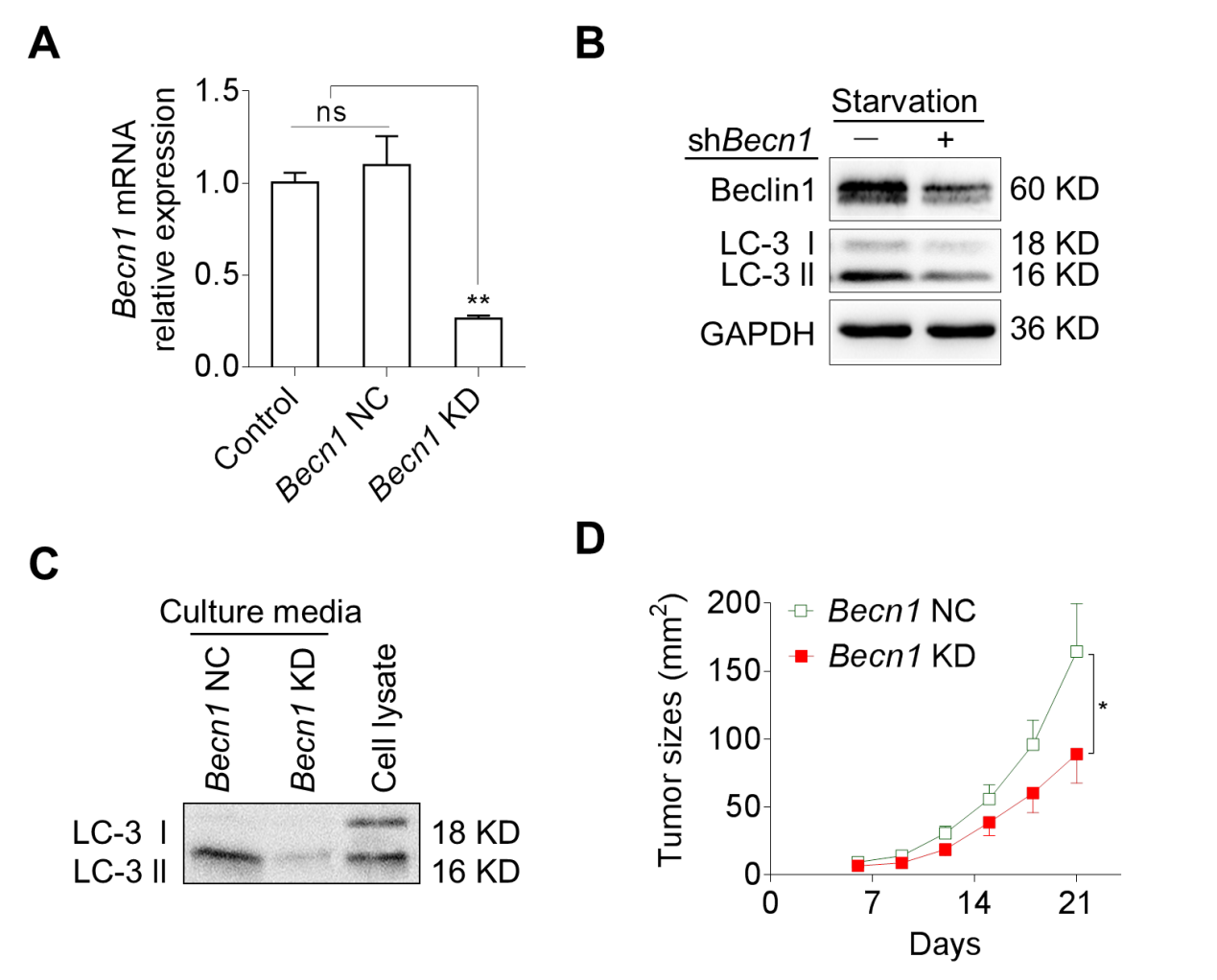


**Figure. S7** Evaluation of Beclin-1 expression in B16F10 cells. **a** The expression of *Becn1* in B16F10 cells after transduction with a lentivirus encoding *Becn1*-shRNA (*Becn1* KD) or scrambled control shRNA (*Becn1* NC) was determined by qRT-PCR. **b** The expression of Beclin-1 and LC-3I/II in B16F10 cells was assessed by western blot. **c** LC-3I/II in the tumor cell culture media from control *Becn1* NC or Beclin1 *Becn1* KD B16F10 cells were detected by western blot. **d** Tumor growth was monitored by calipers. Data (mean ± SEM) represent 3 independent experiments. **, P < 0.01; ns, not significant.

**Figure S8**


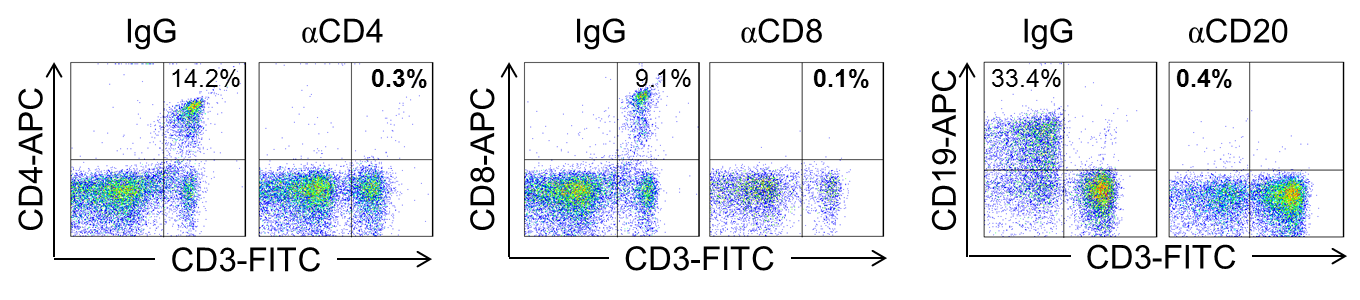


**Figure. S8** Depletion of selected cellular subsets during tumor growth. Groups of C57Bl/6 mice (*n* =5/group) were inoculated with 1×10^6^ *Becn1*-NC or *Becn1*-KD B16F10 cells s.c. in the flank and grown for 9 days**.** For B cells depletion, tumor-bearing mice were injected i.v. with 250 μg of Ultra-LEAF^TM^ purified mAb SA271G2 or isotype control. For CD8^+^ T cells or CD4^+^ T cells depletion, tumor bearing mice were injected i.v. with 250 μg of anti-mouse CD4 (clone GK1.5, BioXCell) or anti-mouse CD8 (clone 2.43, BioXCell) antibodies twice weekly throughout the course of tumor growth. Confirmation of depletions for CD8^+^ T cells, CD4^+^ T cells, and B cells from peripheral blood after antibody administration.
